# Supplementary material for: Early aerial expedition photos reveal 85 years of glacier growth and stability in East Antarctica
Source: Nat Commun. 2024 May 25;15:4466. doi: 10.1038/s41467-024-48886-x (PMC11127979; doi:10.1038/s41467-024-48886-x)
Supplement: Supplementary file 1 — Supplementary Material [file 41467_2024_48886_MOESM1_ESM.pdf]

Early aerial expedition photos reveal 85 years of glacier growth and stability in East Antarctica

Supplementary Material

The 1936/37 Norwegian Antarctic Expedition

The 1936-37 Norwegian Antarctic expedition, also known as the Thorshavn IV expedition (named after the main vessel), was financed by the Norwegian whaling ship owner and consul Lars Christensen, who had special interest in Antarctica. In 1926, he initiated a yearly Antarctic exploration program. His expeditions led to the Norwegian claims of Bouvet Island (1927), Peter I Island (1929), and Dronning Maud Land (1939). Thorshavn IV was Christensen's final expedition, and its specific aim was to capture aerial images for topographic mapping of areas discovered on previous trips.

The expedition included Lars Christensen, his wife Ingrid Christensen, who had become the first woman to reach the Antarctic continent on a previous expedition, and their youngest daughter (Fig. S3, S4, and S6). They departed from Cape Town on December 28th, 1936, on M/T Thorshavn (Fig. S5) and after a short stop at Kerguelen, they arrived at 90° East between Shackleton and the West Ice Shelf, where they had planned to start the mapping. Due to unstable weather conditions, they sailed west, and on January 24th, they completed the first mapping by photographing the West Ice Shelf. From there, they continued westward, and at around 82° East, they started consistently mapping the coast. On February 7th, the expedition had reached its end at Princess Astrid Coast and started its journey home (Fig. S7). Widerøe Airlines was hired for the photogrammetric mapping and the images were captured using a Zeiss camera with a focal length of 210.71 mm and a film format of 18x18 cm[1]. The expedition had an effective flight time of 44 hours, corresponding to a flying distance of 10,000 km. Approximately 2,000 km of the coastline

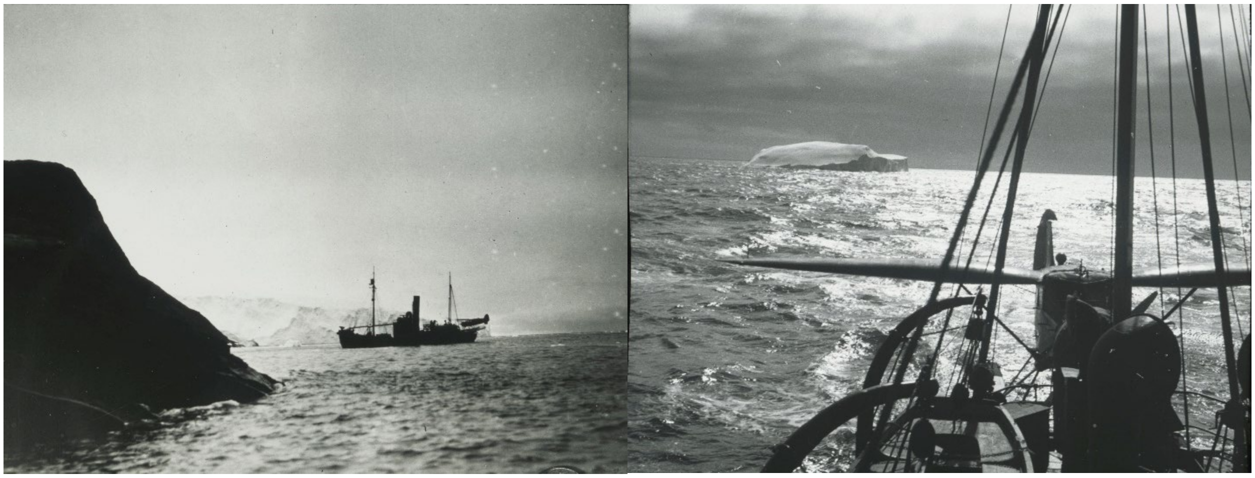

Fig. S1. Whaling ship Firern (loaded with floatplane LN-BAR) captured near Klarius Mikkelsen Fjell in Lars Christensen Land.

was photographed from an altitude of approximately 1400-2000 m a.s.l., resulting in a total of 2,200 aerial images. They used a 1936-model Stinson Reliant floatplane (call sign LN-BAR) chartered from Widerøe Airlines. The aircraft was equipped with a 350 HP Wright engine and an extra fuel tank, giving it a flying range of approximately 1200 km (Fig. S1 and S2). The automatic Zeiss camera, was mounted at the floor of the airplane recording images in a 20° angle below the horizontal plane.

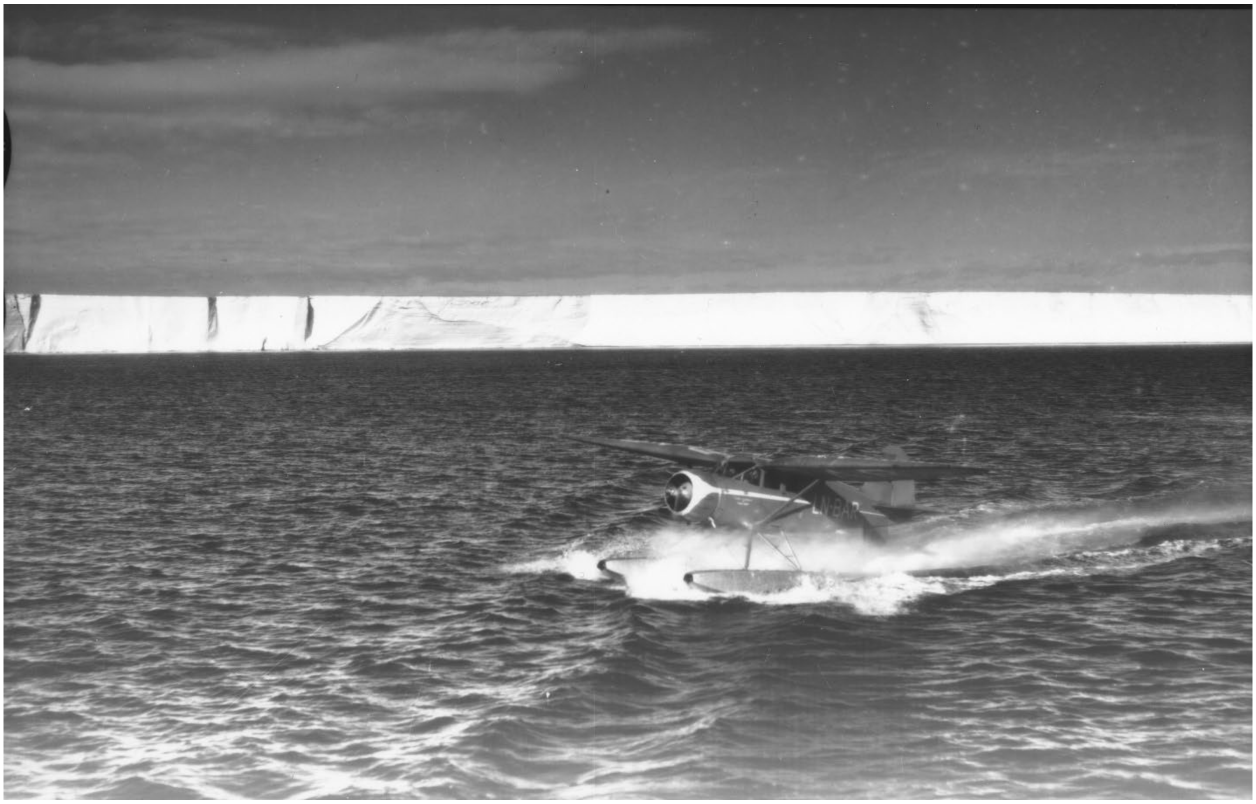

Fig. S2. Stinson Reliant floatplane (call sign LN-BAR) used for aerial photographing.

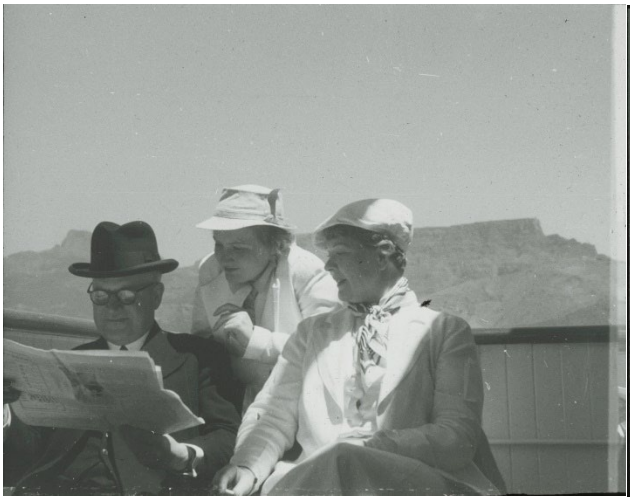

Fig. S3. Lars Christensen, Ingrid Christensen and their daughter onboard M/T Thorshavn outside Cape Town, South Africa.

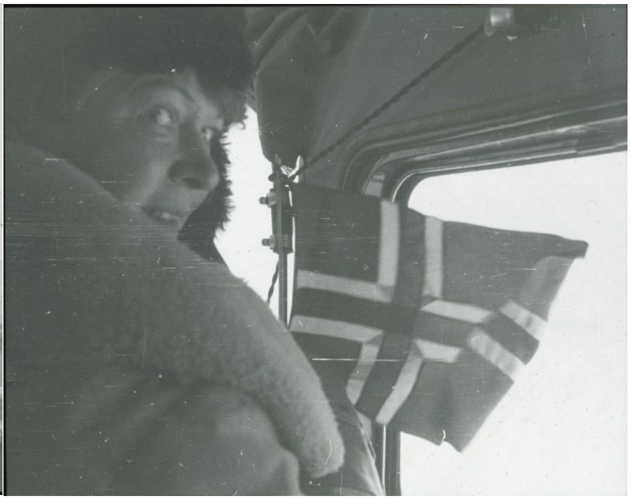

Fig. S4. Ingrid Christensen onboard the floatplane, ready to drop the Norwegian flag near 38° East, 69° South – a region later named Prince Harald Land.

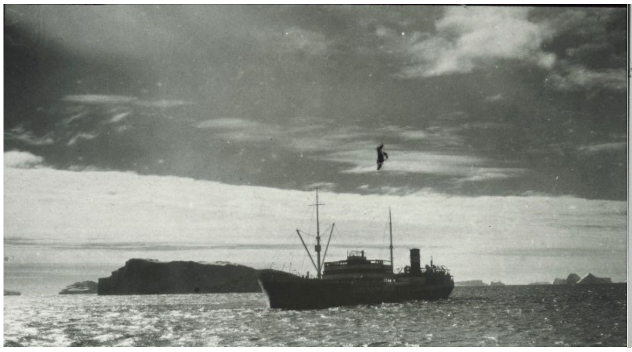

Fig. S5. M/T Thorshavn outside Ingrid Christensen Land.

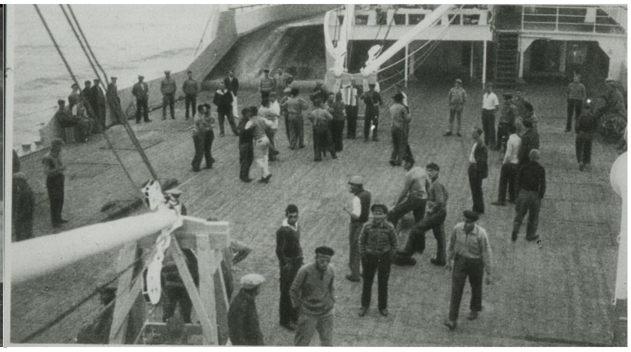

Fig. S6. The crew onboard M/T Thorshavn during the 1936-1937 Antarctic expedition.

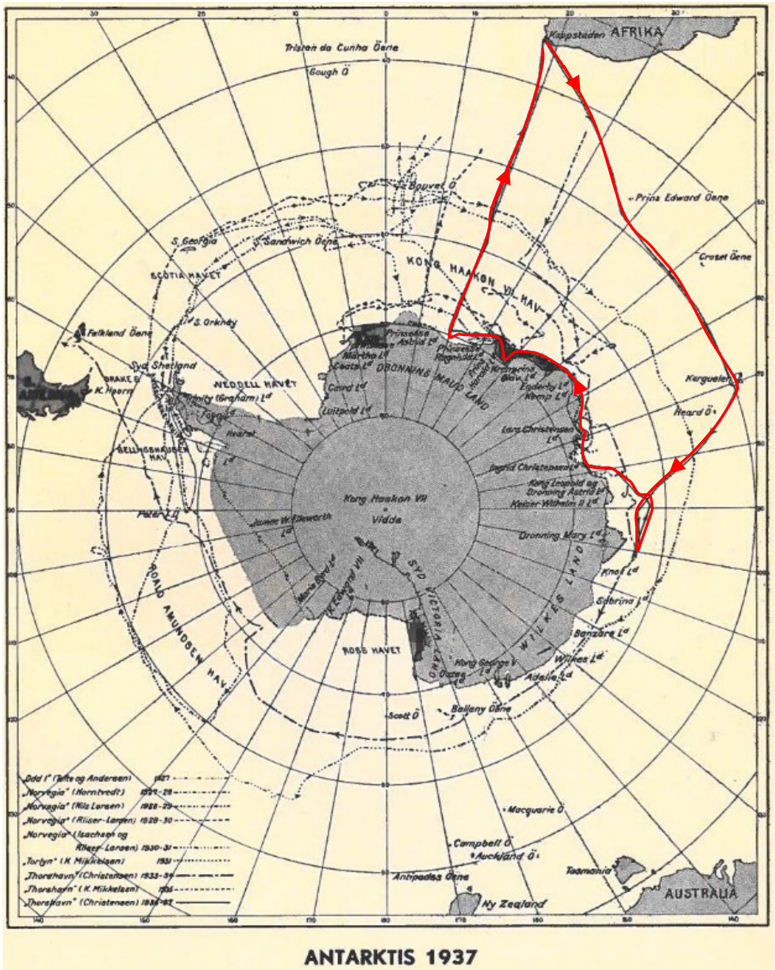

Fig. S7. Map showing all Antarctic expeditions financed by Lars Christensen, adapted from Christensen 1938[1] with the red line showing the 1936/37 expedition route.

Placement of elevation comparison circles

To ensure an optimal positioning of the 150-meter radius elevation comparison circles used for calculating glacier elevation changes, a multi-step approach is employed at each glacier:

First, we convert each historical DEM into a binary dataset, where a value of 0 represents no data and a value of 1 represents the presence of elevation data. Next, we aggregate the binary datasets to generate a composite map that highlights the areas across the glacier with the highest density of historical observations (Fig. S8). To maximize data coverage and ensuring comprehensive representation of the glacier's surface, the circles are positioned in close proximity to each other, extending from the front to the back of the glacier, and targeting areas with a high concentration of historical elevation observations. All circles are positioned within the minimum boundary area defined by the GCPs, to ensure that we include only observations with accurate georeferencing. Moreover, we make efforts to position the circles close to the centerline of the glacier. This is particularly crucial for glaciers that exhibit surface undulations, such as Hoseason and Utstikkar Glacier (Fig. S13 and s15). By adopting this circle placement strategy, we aim to capture the overall characteristics of the glacier, including both the peaks and valleys of these undulations. Surface undulations are formed when the glacier flows over uneven bedrock[2] and as the glacier moves, the undulations also move.

For glaciers that have only one historical DEM, we omit the generation of a composite map. However, we employ a similar approach to position the elevation comparison circles to ensure consistency in our methodology across all glaciers and facilitate easy comparison with the REMA DEM strips, which may also contain areas of data gaps (Fig. S12B and S13C). Due to the data gaps in the historical DEMs, it is not always feasible to adhere to the described positioning approach. This is particularly evident in cases such as Honnörbrygga and Brunvoll Glacier (Fig. S12 and S16), where the circles are positioned across the glacier area instead of close to the centerline. The only exception to this general approach of placing elevation comparison circles is Brown Glacier (Fig. S19), where elevation changes are calculated across the entire glacier area as the historical DEM contains no void areas.

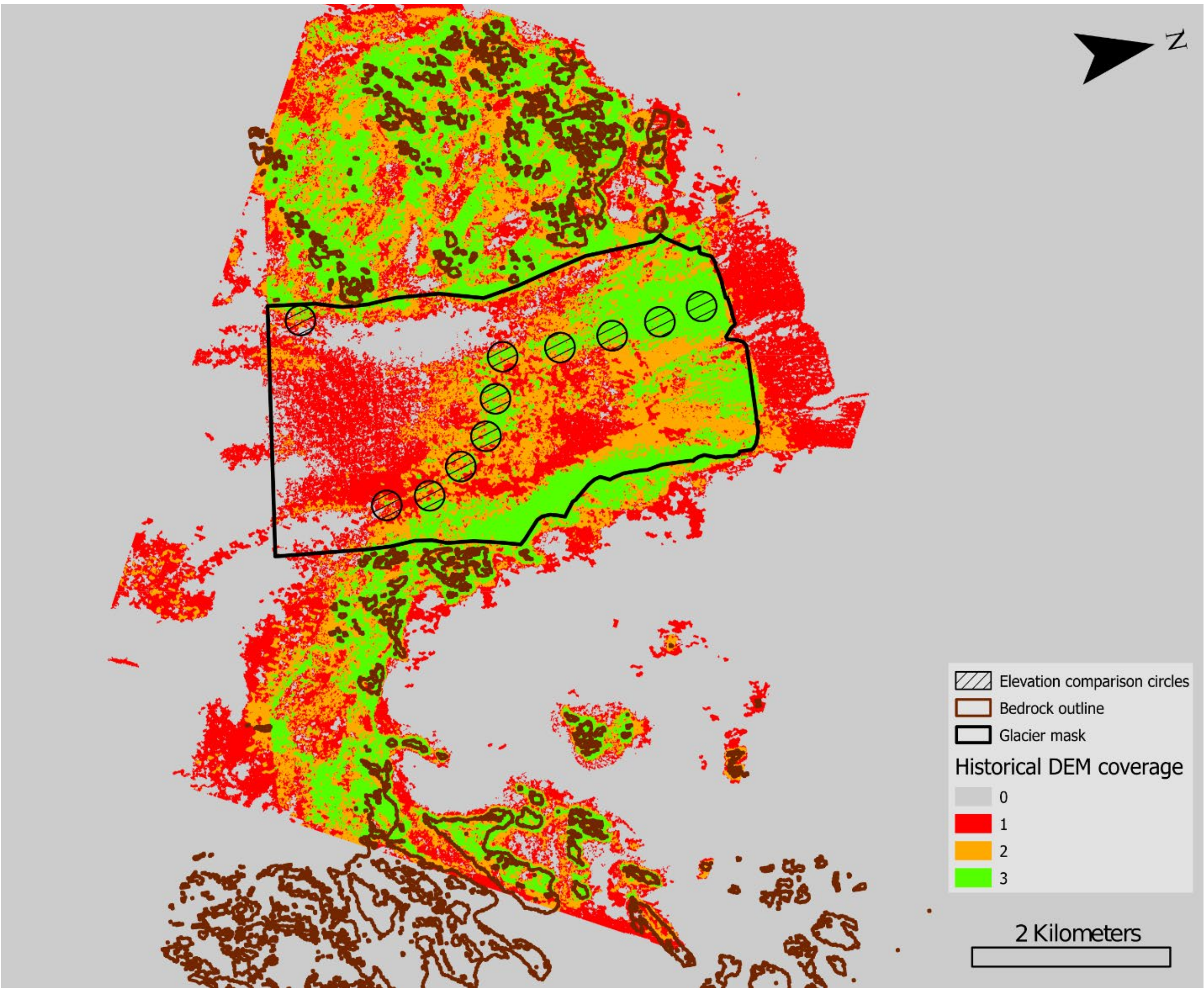

Fig. S8. Composite map of the 1937, 1956, and 1973 historical DEM coverage at Taylor Glacier. Each pixel color indicates the number of historical observations, to identify the areas across the glacier with the highest density of historical observations.

Sensitivity analysis of elevation changes

To evaluate the impact of circle placement on the mean dH values for each period and glacier, we perform sensitivity tests. We calculate the mean dH of all combinations of N/2 circles (N being the total number of circles) and determine the standard deviation (std) across all these combinations (Fig. S9). To account for the sampling uncertainty introduced by circle placement, we adopt two times the standard deviation (2*std) as an additional uncertainty when interpreting the mean dH obtained using all circles (Fig 2). Notably, we observe that glaciers with large surface undulations (e.g. Hoseason Gl.) exhibited larger sampling uncertainty compared to the estimated model uncertainty of our generated DEMs. Conversely, glaciers with a more uniform surface (e.g. Honnörbrygga Gl.) show smaller sampling uncertainty (Fig 2).

To further assess the sensitivity of our results, we also perform a positional shift analysis by slightly adjusting the positions of the circles and examining the resulting impact on the calculated dH values. However, it is important to note that due to data gaps in the historical DEMs, we are unable to conduct this analysis for all glaciers. Therefore, we specifically select two types of glaciers with good historical data coverage for this analysis: Utstikkar Glacier, characterized by substantial surface undulations, and Honnörbrygga Glacier, which exhibits a relatively smooth surface. For Honnörbrygga Glacier the circles are shifted approximately 150 m in front of the initial position (Fig S10A.). On the other hand, for Utstikkar Glacier, the circles are shifted across the glacier area while attempting to maintain a consistent distance from the glacier front (Fig. S10B). However, due to data gaps, it is not possible to shift the circle farthest from the front. For Hönnorbrygga Glacier, we calculate the mean dH of all 257 combinations of circles, whereas for Utstikkar Glacier the mean dH is based on 1000 random combinations. We exclude combinations that involved both the original and shifted versions of the same circle, eliminating any duplication. Next, we compare the mean dH from the shifted circle combinations with the dH from the initial placement and find a limited effect on the dH results. Specifically, at Honnörbrygga Glacier, we observe a difference of 0.9 m for the period 1937-2016 and 0.2 m for the period 2016-2020, giving a total difference of 1.1 m from 1937 to 2020. For Utstikkar Glacier, the shifted mean dH is slightly smaller than the original dH estimate, with difference in dH ranging from 0.4 m to 1.2 m across all periods, leading to a total dH difference of 1.4m. To account for this sampling issue, we add an additional conservative uncertainty of 1.5 m on top of the existing sampling uncertainty (Fig. 2). These findings provide confidence that the mean dH determined from the initial circle placement represents the changes occurring across the glacier and provides a more comprehensive assessment of the overall reliability of our results.

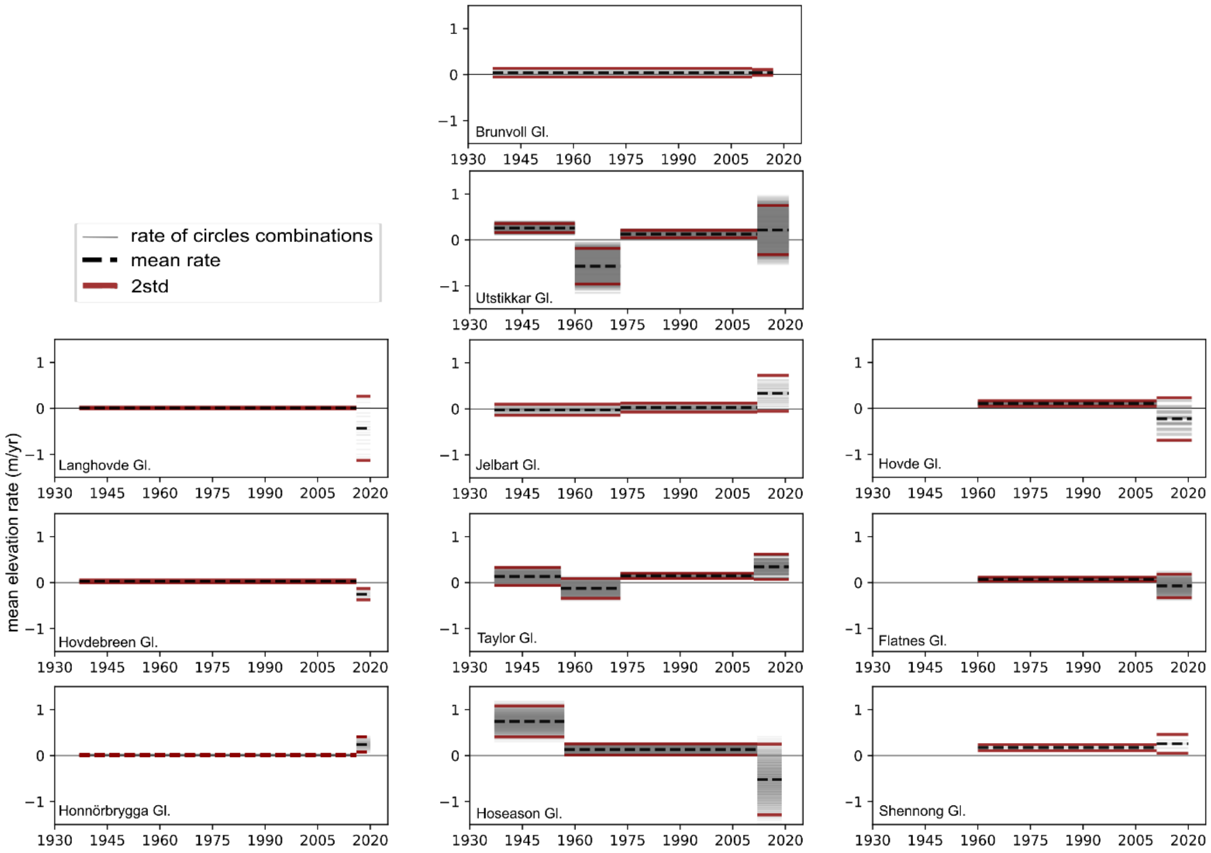

Fig. S9. Sensitivity analysis of all glacier. The sensitivity is determined by calculating the mean rate of all
115 **combinations of N/2 circles, the 2*std across all these combinations, and the mean rate.**

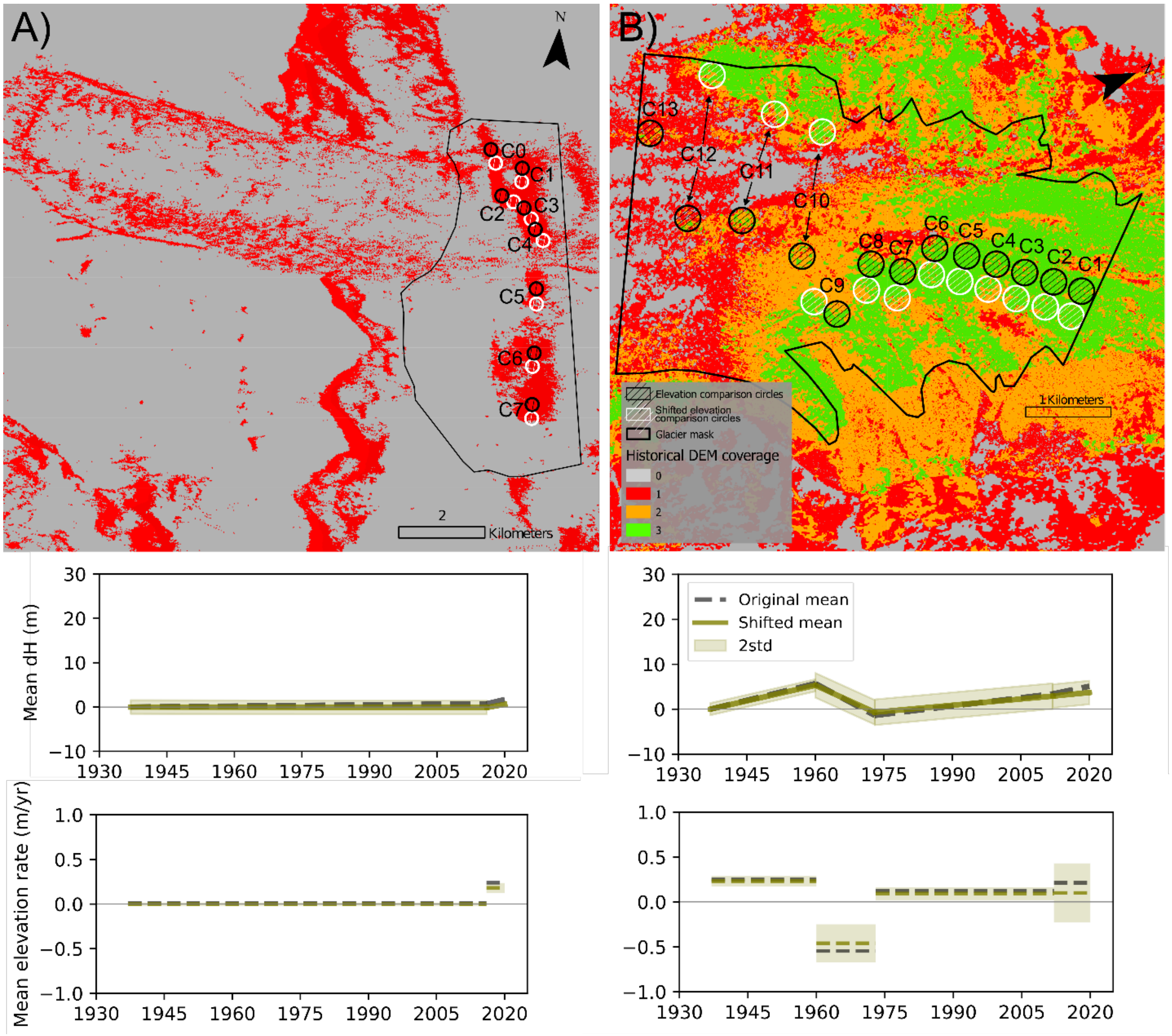

Figure S10. Composite map of historical DEM coverage and plot showing a comparison of the original mean and the shifted mean shown as absolute dH and elevation rate (calculated based on all possible combinations of circles) for A) Honnörbrygga Glacier and B) Utstikkar Glacier.

Table S1. Overview of all glaciers included in the study and their historical observations.

Region name	Glacier name	Historical frontal position	Historical ice thickness	Historical velocity
Lützow-Holm Bay	Honnörbrygga	X	X	
Lützow-Holm Bay	Hovdebreen	X	X	
Lützow-Holm Bay	Langhovde	X	X	
Lützow-Holm Bay	Telen	X		
Lützow-Holm Bay	Skallen	X		
Lützow-Holm Bay	Shirase	X		
Kemp and Mac Robertson Land	Hoseason	X	X	X
Kemp and Mac Robertson Land	Taylor	X	X	X
Kemp and Mac Robertson Land	Jelbart	X	X	X
Kemp and Mac Robertson Land	Utstikkar	X	X	X
Kemp and Mac Robertson Land	Brunvoll	X	X	
Kemp and Mac Robertson Land	Unnamed	X		
Kemp and Mac Robertson Land	Forbes	X		
Kemp and Mac Robertson Land	Mulebreen	X		
Ingrid Christensen Coast	Shennong	X	X	
Ingrid Christensen Coast	Flatnes	X	X	
Ingrid Christensen Coast	Hovde	X	X	
Ingrid Christensen Coast	Brown	X	X	
Ingrid Christensen Coast	Dolk	X		
Ingrid Christensen Coast	Chaos	X		
Ingrid Christensen Coast	Sørdals	X		

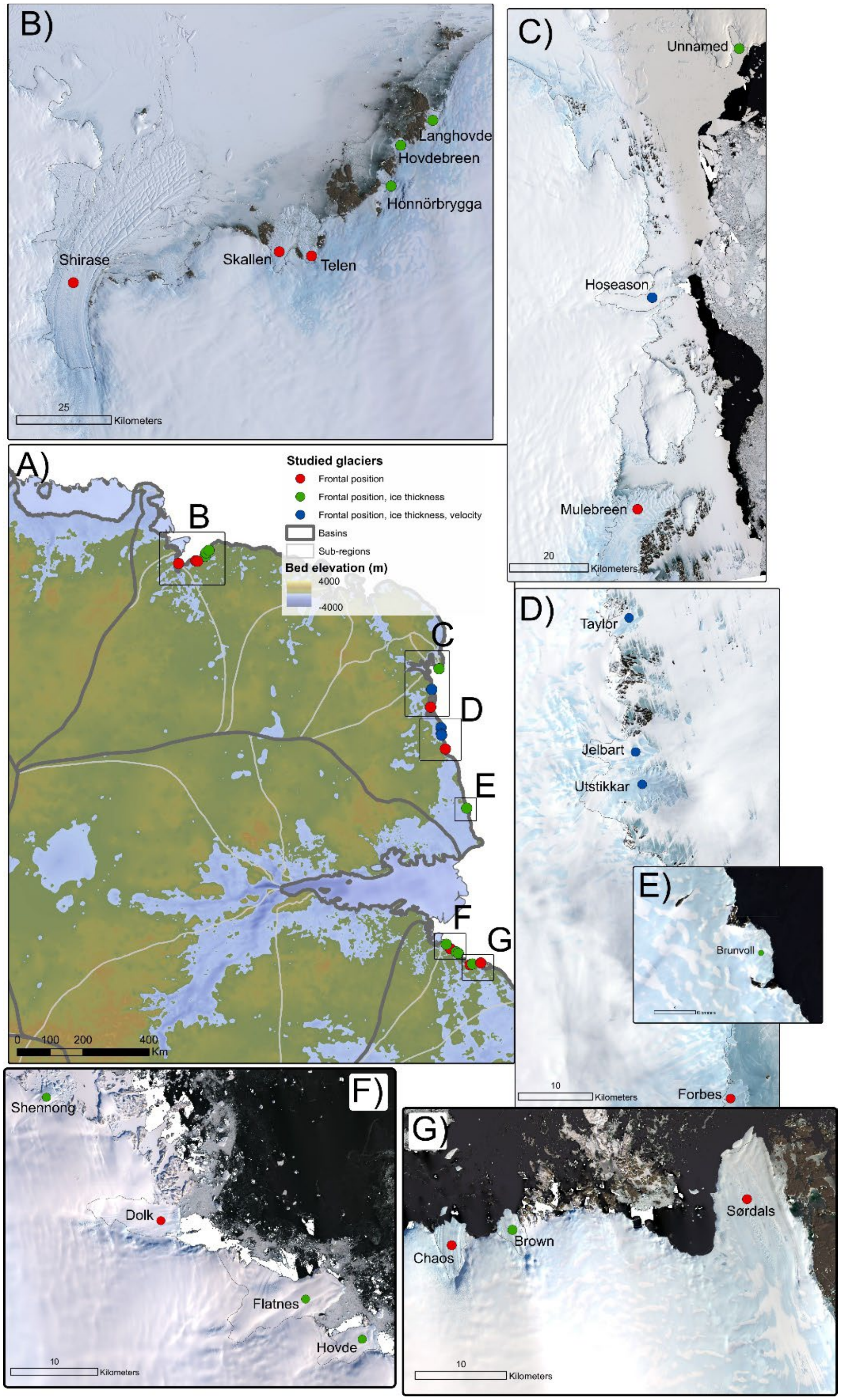

Fig. S11. Overview of all glaciers included in the study and their historical observations. The overview map A) also show basins and sub-regions. Panels B), C), D), E), F), and G) show close-ups of all studied glaciers on Landsat scenes from 2022-2023.

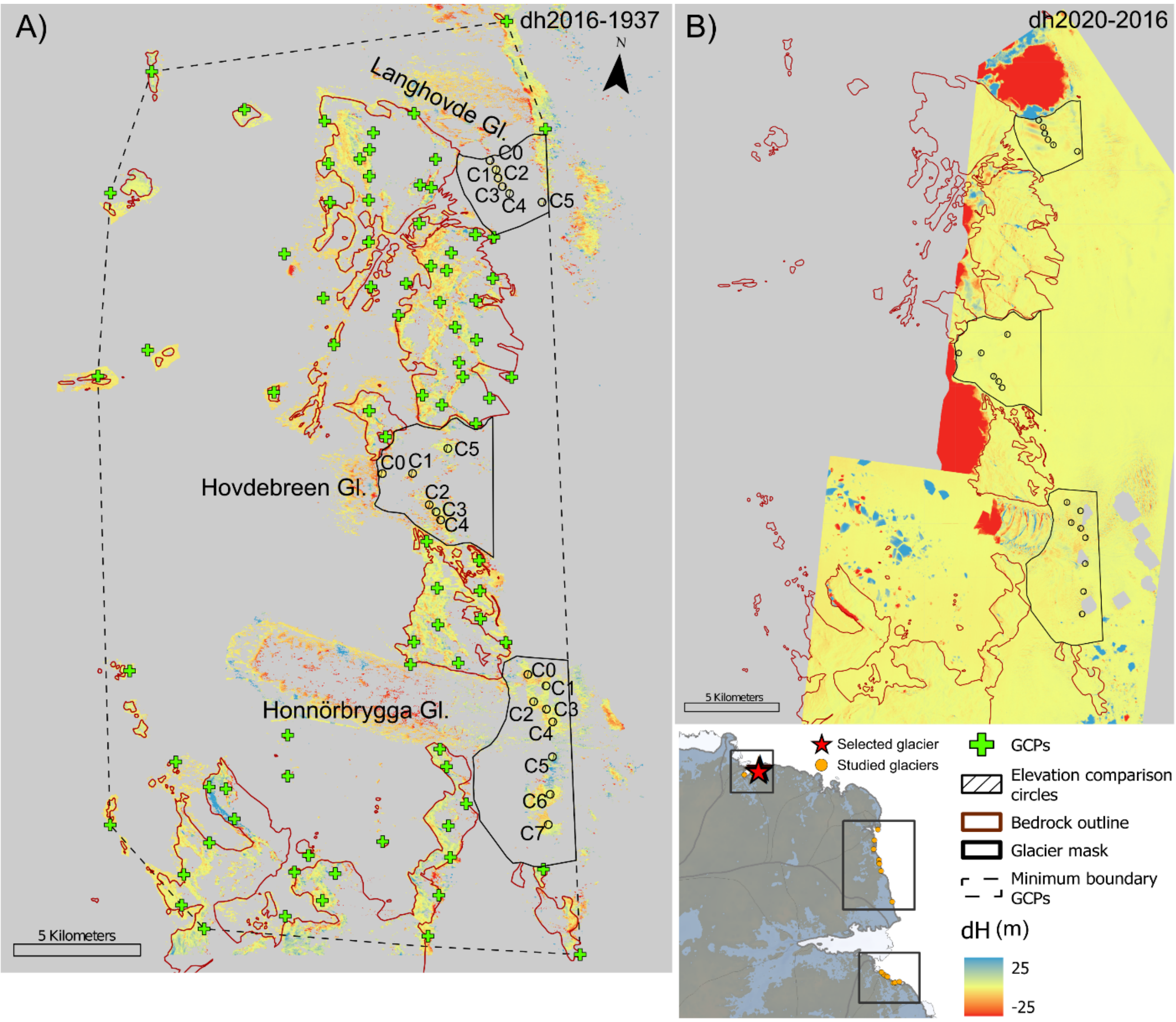

Langhovde Gl.

C)

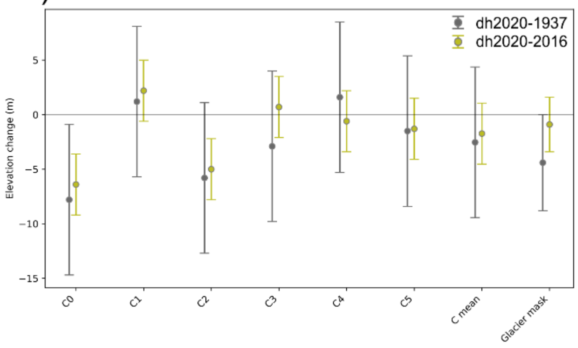

D)

Elevation comparison area	dh2020-1937 (m)	err2020-1937 (m)	dh2020-2016 (m)	err2020-2016 (m)
C0	-7.8	7.0	-6.4	3.0
C1	1.2	7.0	2.2	3.0
C2	-5.8	7.0	-5	3.0
C3	-2.9	7.0	0.7	3.0
C4	1.6	7.0	-0.6	3.0
C5	-1.5	7.0	-1.3	3.0
C-mean	-2.5	7.0	-1.7	3.0
Glacier mask mean	-4.4	4.5	-0.9	2.7

Hovdebreen Gl.

E)

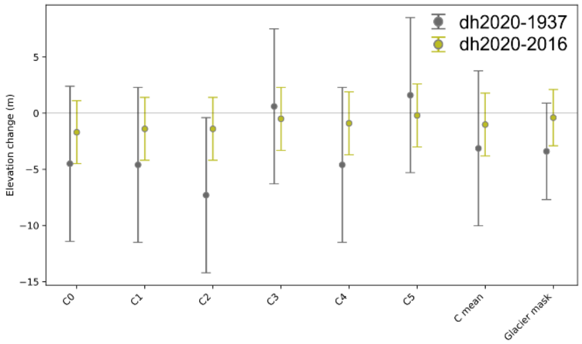

F)

Elevation comparison area	dh2020-1937 (m)	err2020-1937 (m)	dh2020-2016 (m)	err2020-2016 (m)
C0	-4.5	7.0	-1.7	3.0
C1	-4.6	7.0	-1.4	3.0
C2	-7.3	7.0	-1.4	3.0
C3	0.6	7.0	-0.5	3.0
C4	-4.6	7.0	-0.9	3.0
C5	1.6	7.0	-0.2	3.0
C-mean	-3.1	7.0	-1.0	3.0
Glacier mask mean	-3.4	4..4	-0.4	2.7

Honnörbrygga Gl.

G)

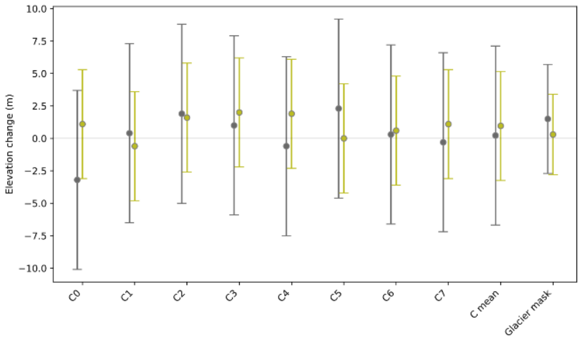

H)

Elevation comparison area	dh2020-1937 (m)	err2020-1937 (m)	dh2020-2016 (m)	err2020-2016 (m)
C0	-3.2	7.0	1.1	4.3
C1	0.4	7.0	-0.6	4.3
C2	1.9	7.0	1.6	4.3
C3	1.0	7.0	2.0	4.3
C4	-0.6	7.0	1.9	4.3
C5	2.3	7.0	0.0	4.3
C6	0.3	7.0	0.6	4.3
C7	-0.3	7.0	1.1	4.3
C-mean	0.2	7.0	1.0	4.3
Glacier mask mean	1.5	4.3	0.3	3.3

I)

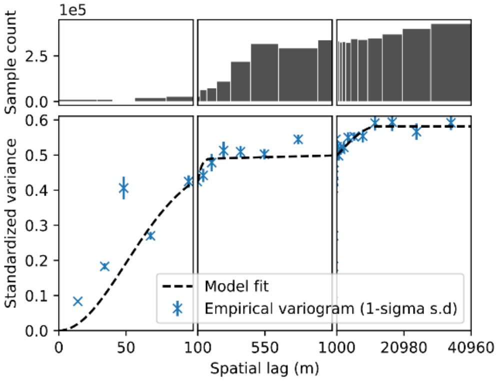

Fig. S12. Elevation changes at Langhovde, Hovdebreen, and Honnörbrygga Glacier A) 2016-1937 and B) 2020-2016. Elevation changes and associated errors of elevation comparison circle and the glacier area shown as C), E), and G) scatterplot as D), F) and H) tables for Langhovde Gl., Hovdebreen Gl. and Honnörbrygga Gl., respectively. I) Spatial variogram of the 1937 historical DEM. Note that A) shows the dH2016-1937, whereas the scatterplots and tables show the dH2020-1937, as no 2020 reference DEM strips covered the entire SfM-model area.

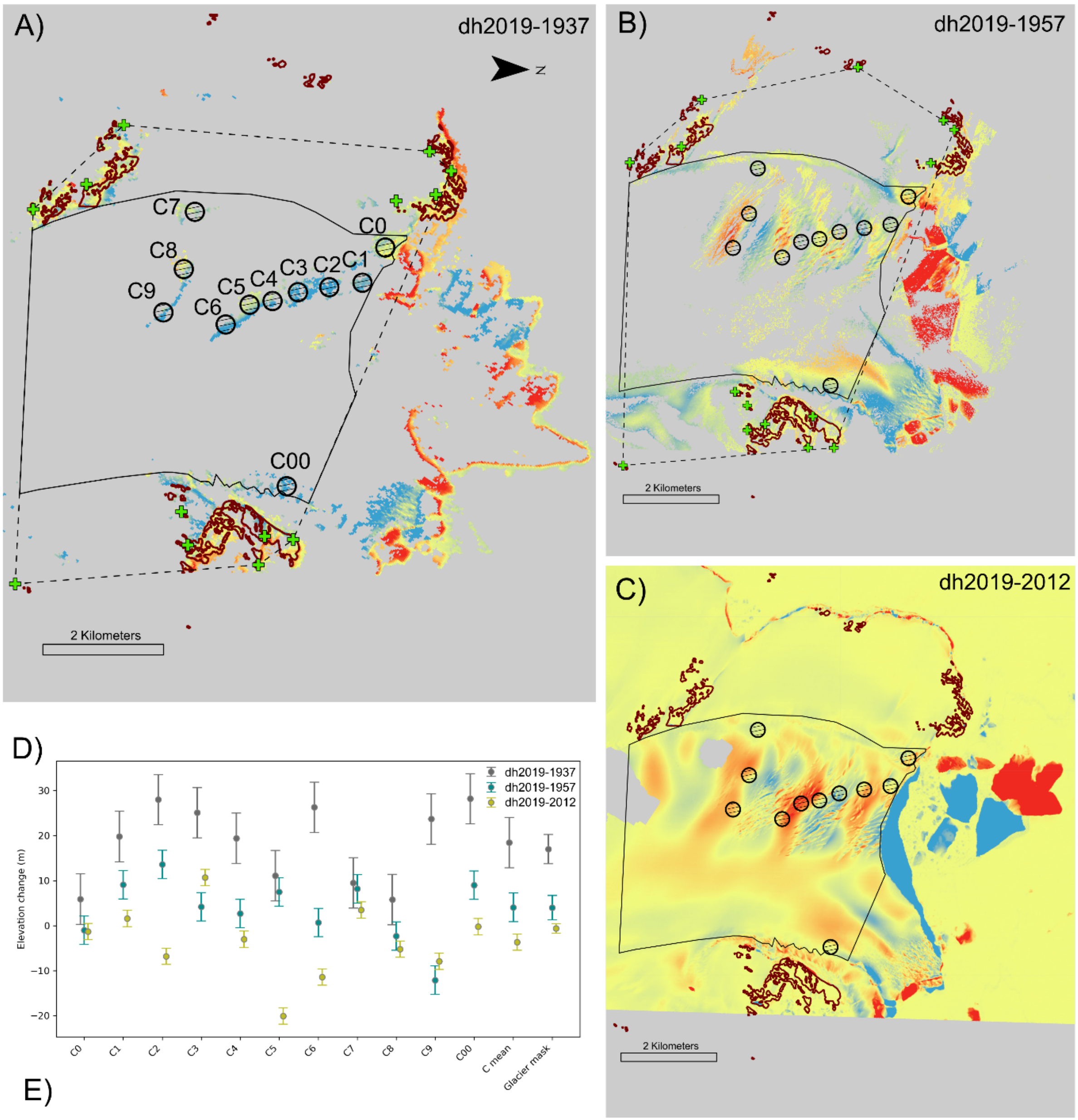

E)

Elevation comparison area	dh2019-1937 (m)	err2019-1937 (m)	dh2019-1957 (m)	err2019-1957 (m)	dh2019-2012 (m)	err2019-2012 (m)
C0	5.9	5.6	-1.0	3.2	-1.3	1.8
C1	19.8	5.6	9.1	3.2	1.6	1.8
C2	28	5.6	13.6	3.2	-6.8	1.8
C3	25.1	5.6	4.2	3.2	10.7	1.8
C4	19.4	5.6	2.7	3.2	-3.0	1.8
C5	11.1	5.6	7.5	3.2	-20.1	1.8
C6	26.3	5.6	0.7	3.2	-11.4	1.8
C7	9.5	5.6	8.2	3.2	3.5	1.8
C8	5.8	5.6	-2.3	3.2	-5.2	1.8
C9	23.7	5.6	-12.1	3.2	-7.9	1.8
C00	28.2	5.6	9.0	3.2	-0.2	1.8
C-mean	18.4	5.6	4.1	3.2	-3.6	1.8
Glacier mask mean	17.0	3.3	4.0	2.7	-0.6	1.0

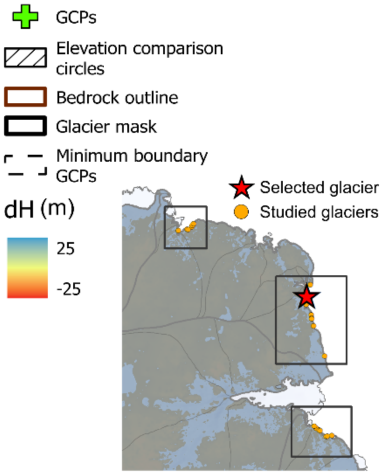

F)

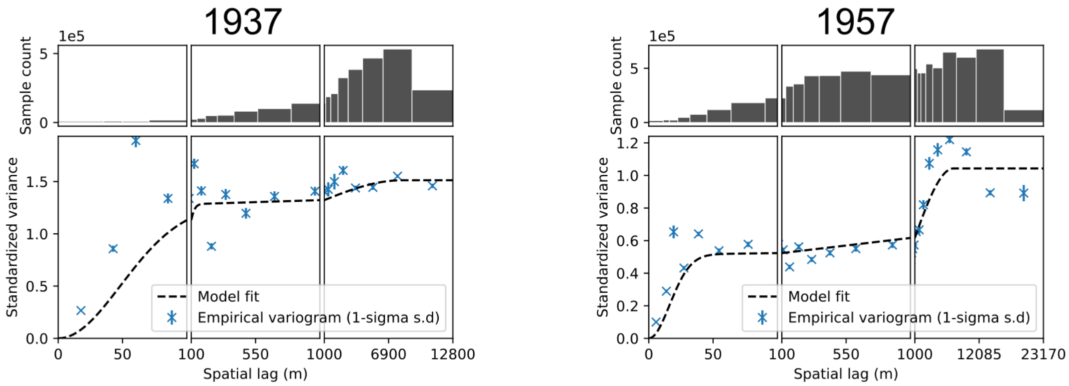

Fig. S13. Elevation changes at Hoseason Glacier A) 2019-1937, B) 2019-1957, C) 2019-2012. Elevation changes and associated errors of elevation comparison circle and the glacier area shown as D) scatterplot and E) table. F) Spatial variogram of the 1937 and 1957 historical DEM

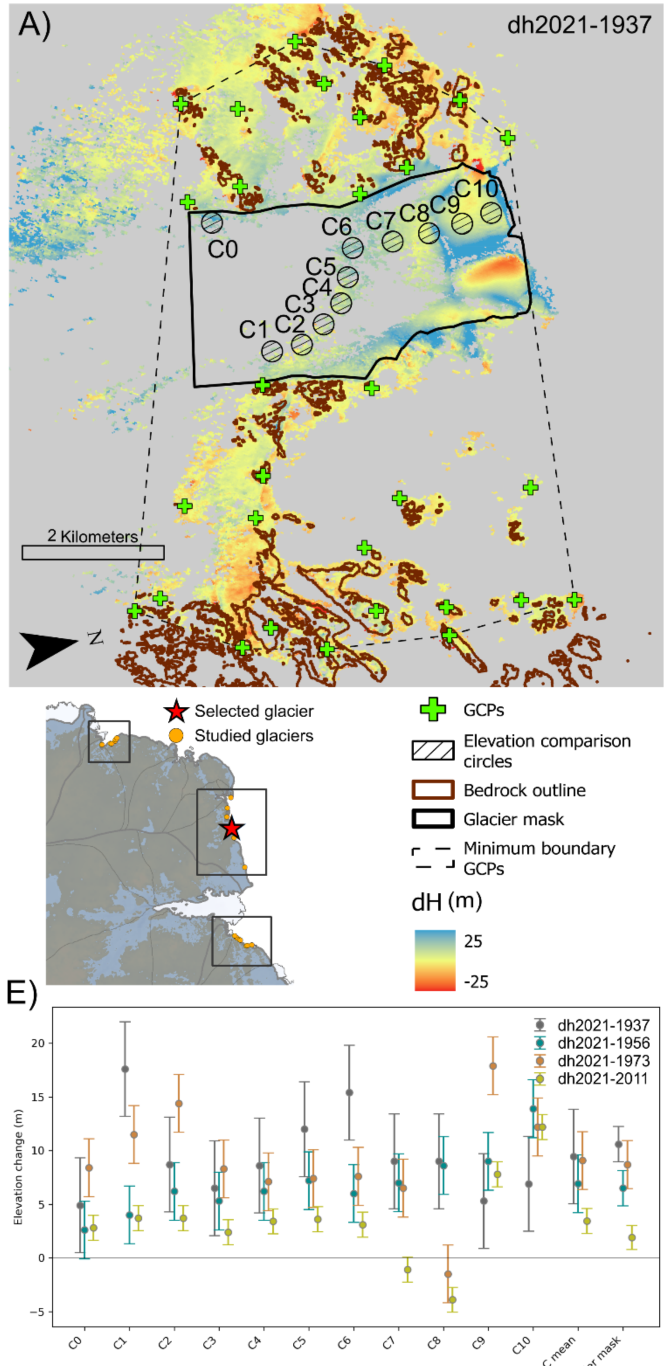

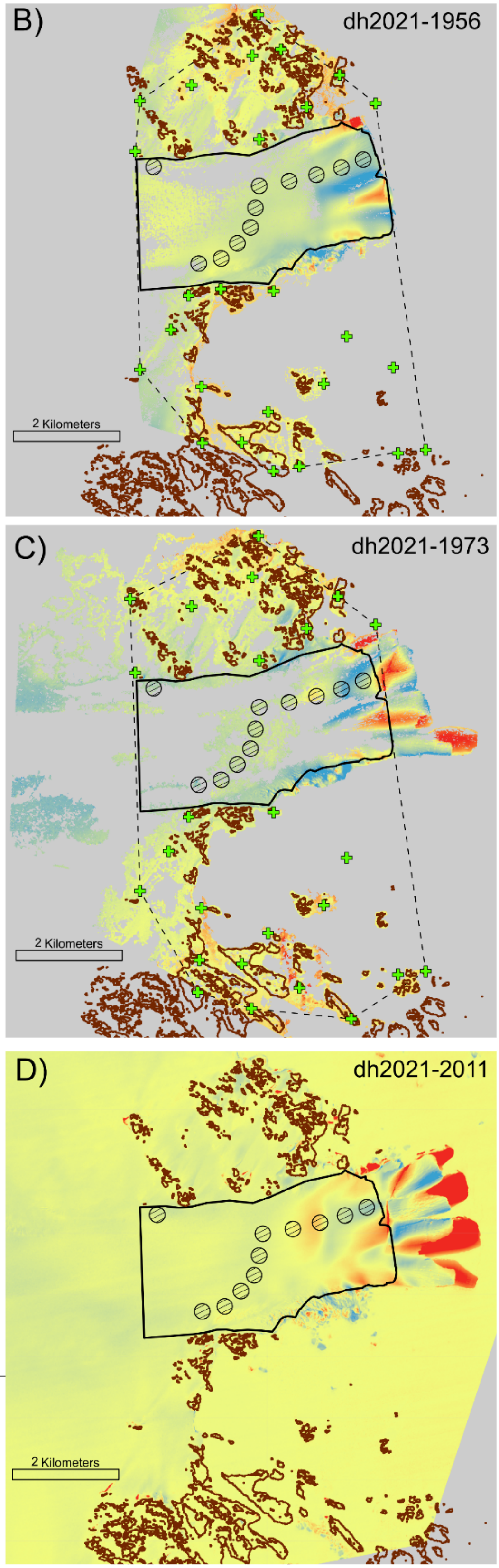

F)

Elevation comparison area	dh2021-1937 (m)	err2021-1937 (m)	dh2021-1956 (m)	err2021-1956 (m)	dh2021-1973 (m)	err2021-1973 (m)	dh2021-2011 (m)	err2021-2011 (m)
C0	4.9	4.4	2.6	2.7	8.4	2.7	2.8	1.2
C1	17.6	4.4	4	2.7	11.5	2.7	3.7	1.2
C2	8.7	4.4	6.2	2.7	14.4	2.7	3.7	1.2
C3	6.5	4.4	5.3	2.7	8.3	2.7	2.4	1.2
C4	8.6	4.4	6.2	2.7	7.1	2.7	3.4	1.2
C5	12.0	4.4	7.2	2.7	7.4	2.7	3.6	1.2
C6	15.4	4.4	6	2.7	7.6	2.7	3.1	1.2
C7	9.0	4.4	7	2.7	6.5	2.7	-1.1	1.2
C8	9.0	4.4	8.6	2.7	-1.5	2.7	-3.9	1.2
C9	5.3	4.4	9	2.7	17.9	2.7	7.8	1.2
C10	6.9	4.4	13.9	2.7	12.2	2.7	12.2	1.2
C-mean	9.4	4.4	6.9	2.7	9.1	2.7	3.4	1.2
Glacier mask mean	10.6	1.6	6.5	1.6	8.7	1.9	1.9	1.1

G)

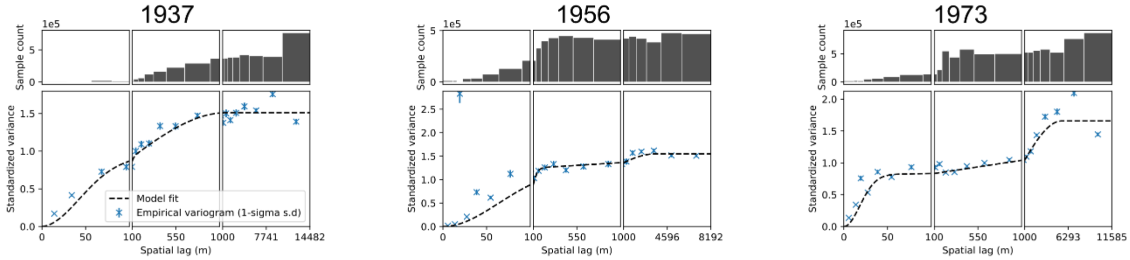

Fig. S14. Elevation changes at Taylor Glacier A) 2021-1937, B) 2021-1956, C) 2021-1973, and D) 2021-2011. Elevation changes and associated errors of elevation comparison circle and the glacier area shown as E) scatterplot and F) table. G) Spatial variograms for each of the historical DEMs

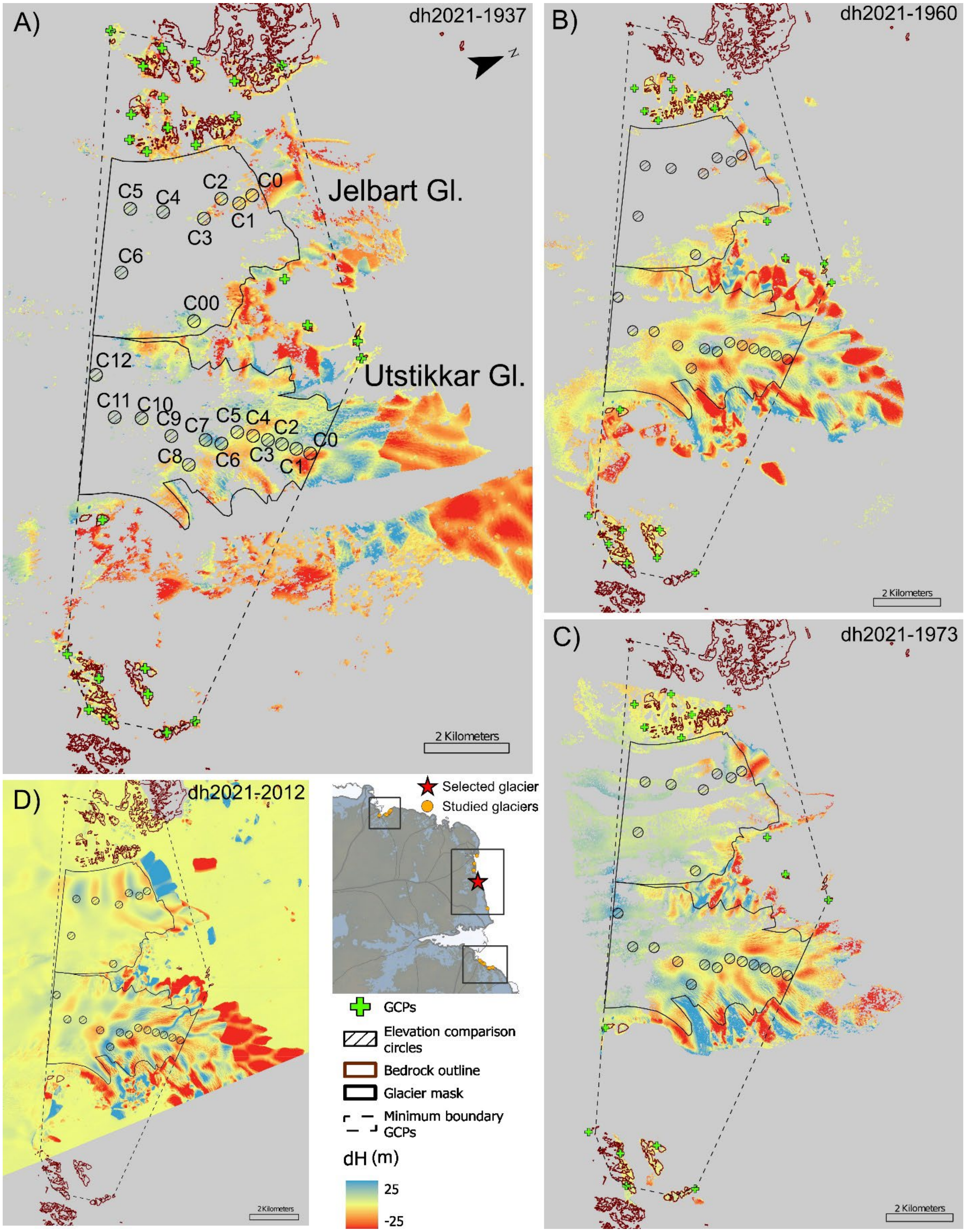

A)
dh2021-1937
N
Jelbart Gl.
Utstikkar Gl.
C0
C1
C2
C3
C4
C5
C6
C00
C12
C11
C10
C9
C8
C7
2 Kilometers
B)
dh2021-1960
2 Kilometers
C)
dh2021-1973
2 Kilometers
D)
dh2021-2012
2 Kilometers
Selected glacier
Studied glaciers
GCPs
Elevation comparison circles
Bedrock outline
Glacier mask
Minimum boundary GCPs
dH (m)
25
-25

Jelbart Gl.

E)

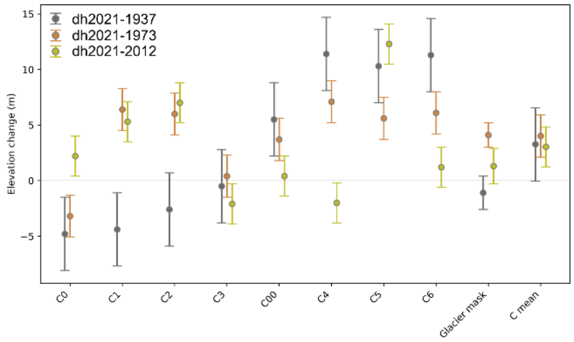

F)

Elevation comparison area	dh2021-1937 (m)	err2021-1937 (m)	dh2021-1973 (m)	dh2021-1973 (m)	dh2021-2012 (m)	err2021-2012 (m)
C0	-4.8	3.4	-3.2	2.1	2.2	2.1
C1	-4.4	3.4	6.4	2.1	5.3	2.1
C2	-2.6	3.4	6	2.1	7	2.1
C3	-0.5	3.4	0.4	2.1	-2.1	2.1
C00	5.5	3.4	3.7	2.1	0.4	2.1
C4	11.4	3.4	7.1	2.1	-2	2.1
C5	10.3	3.4	5.6	2.1	12.3	2.1
C6	11.3	3.4	6.1	2.1	1.2	2.1
C-mean	3.3	3.4	4.0	2.1	3.0	2.1
Glacier mask mean	-1.1	1.8	4.1	1.5	1.3	1.9

Utstikkar Gl.

G)

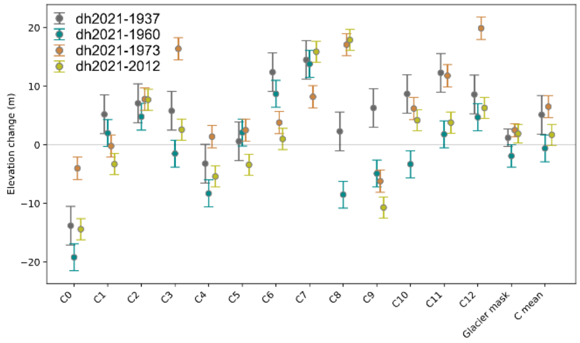

H)

Elevation comparison area	dh2021-1937 (m)	err2021-1937 (m)	dh2021-1960 (m)	err2021-1960 (m)	dh2021-1973 (m)	err2021-1973 (m)	dh2021-2012 (m)	err2021-2012 (m)
C0	-13.8	3.4	-19.2	2.5	-4	2.1	-14.4	2.1
C1	5.2	3.4	2	2.5	-0.2	2.1	-3.3	2.1
C2	7.1	3.4	4.8	2.5	7.8	2.1	7.7	2.1
C3	5.8	3.4	-1.5	2.5	16.4	2.1	2.6	2.1
C4	-3.2	3.4	-8.3	2.5	1.4	2.1	-5.4	2.1
C5	0.6	3.4	2.1	2.5	2.5	2.1	-3.4	2.1
C6	12.4	3.4	8.7	2.5	3.8	2.1	1	2.1
C7	14.5	3.4	13.8	2.5	8.2	2.1	15.9	2.1
C8	2.3	3.4	-8.5	2.5	17.1	2.1	17.9	2.1
C9	6.3	3.4	-4.9	2.5	-6.2	2.1	-10.7	2.1
C10	8.7	3.4	-3.3	2.5	6.2	2.1	4.2	2.1
C11	12.3	3.4	1.8	2.5	11.8	2.1	3.8	2.1
C12	8.6	3.4	4.7	2.5	19.9	2.1	6.3	2.1
C-mean	5.1	3.4	-0.6	2.5	6.5	2.1	1.7	2.1
Glacier mask mean	1.2	1.8	-1.9	2.1	2.5	1.5	1.9	1.9

I)

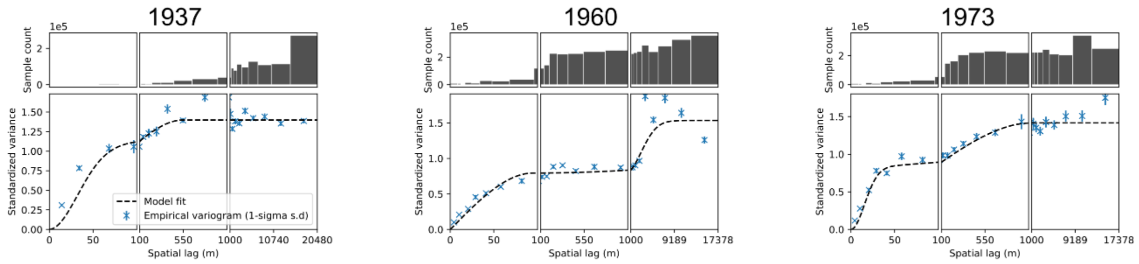

Fig. S15. Elevation changes at Jelbart and Utstikkar Glacier A) 2021-1937, B) 2021-1960, C) 2021-1973, and D) 2021-2012. Elevation changes and associated errors of elevation comparison circle and the glacier area shown as E) and G) scatterplots and F) and H) tables for Jelbart Gl. and Utstikkar Gl., respectively. I) Spatial variograms for each of the historical DEMs. Note that for Jelbart Gl. we do not include data from the 1960 DEM, due to poor coverage.

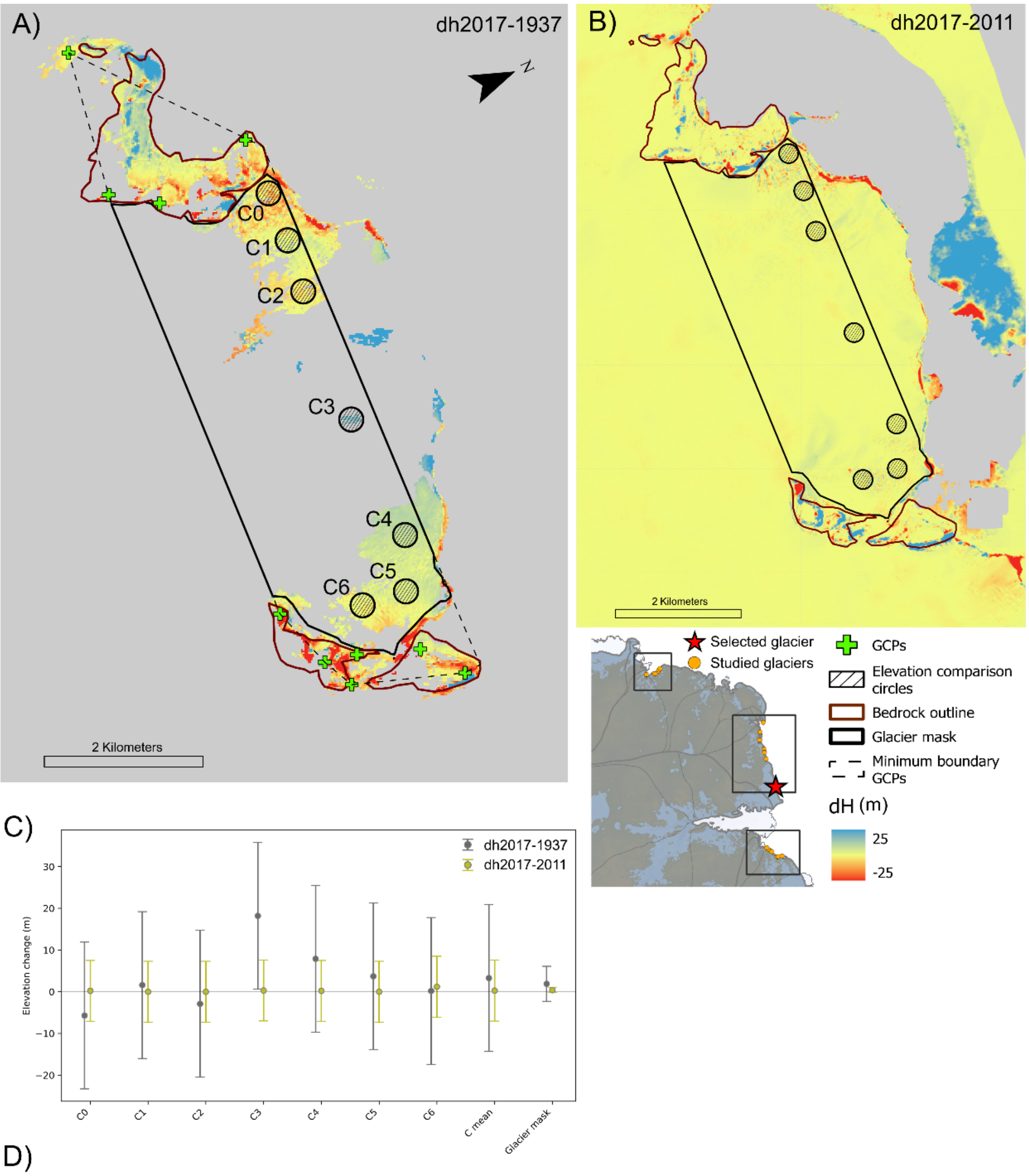

D)

Elevation comparison area	dh2017-1937 (m)	err2021-1937 (m)	dh2017-2011 (m)	err2017-2011 (m)
C0	-5.7	17.6	0.2	7.4
C1	1.6	17.6	0.0	7.4
C2	-2.9	17.6	0.0	7.4
C3	18.2	17.6	0.3	7.4
C4	7.9	17.6	0.2	7.4
C5	3.7	17.6	0.0	7.4
C6	0.2	17.6	1.2	7.4
C-mean	3.3	17.6	0.3	7.4
Glacier mask mean	1.9	4.2	0.4	1.2

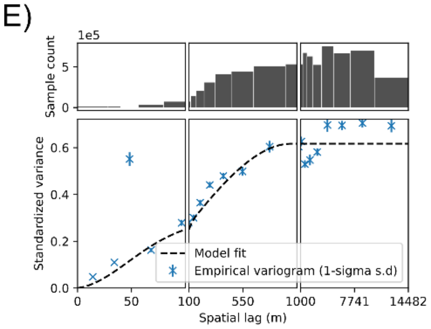

Fig. S16. Elevation changes at Brunvoll Glacier A) 2017-1937, B) 2017-2011. Elevation changes and associated errors of elevation comparison circle and the glacier area shown as C) scatterplot and D) table. E) Spatial variogram of the historical DEM.

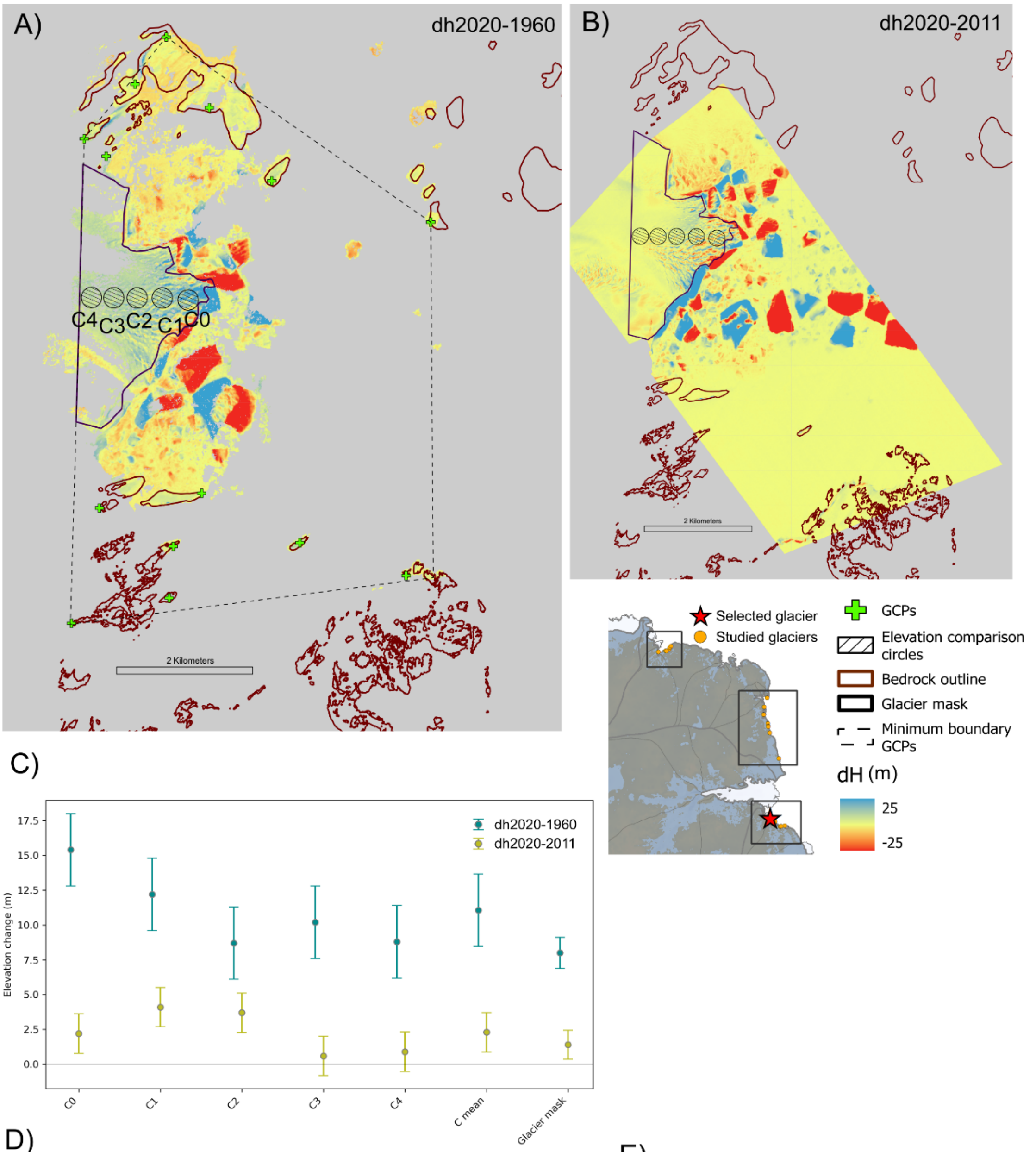

D)

Elevation comparison area	dh2020-1960 (m)	err2020-1960 (m)	dh2020-2011 (m)	err2020-2011 (m)
C0	15.4	2.6	2.2	1.4
C1	12.2	2.6	4.1	1.4
C2	8.7	2.6	3.7	1.4
C3	10.2	2.6	0.6	1.4
C4	8.8	2.6	0.9	1.4
C-mean	11.1	2.6	2.3	1.4
Glacier mask mean	8.0	1.1	1.4	1.0

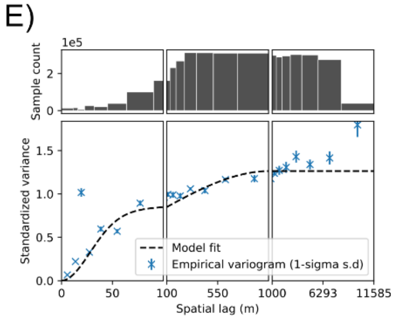

Fig. S17. Elevation changes at Shennong Glacier A) 2020-1960, B) 2020-2011. Elevation changes and associated errors of elevation comparison circle and the glacier area shown as C) scatterplot and D) table. E) Spatial variogram of the historical DEM.

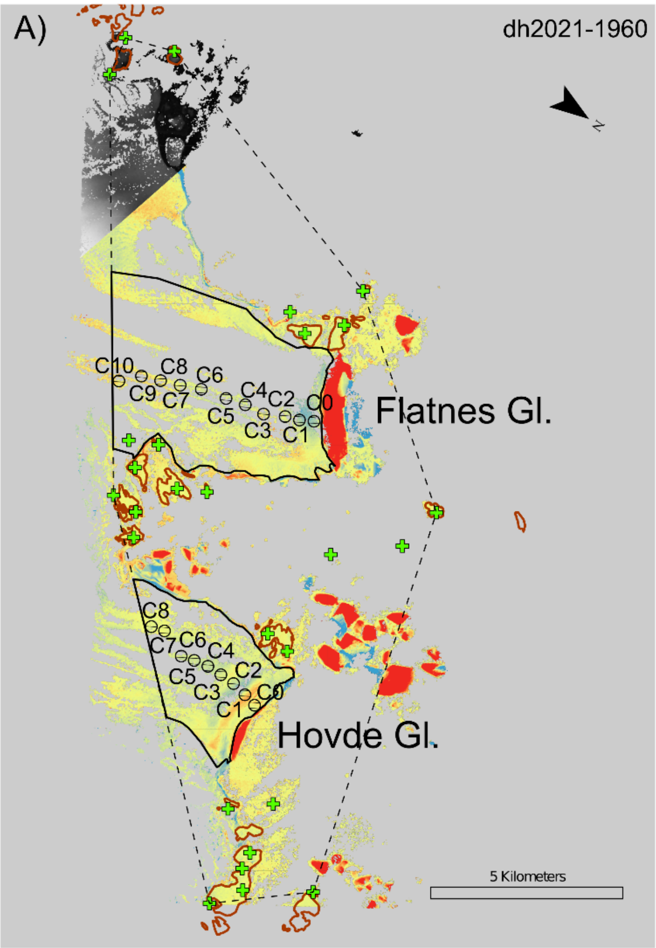

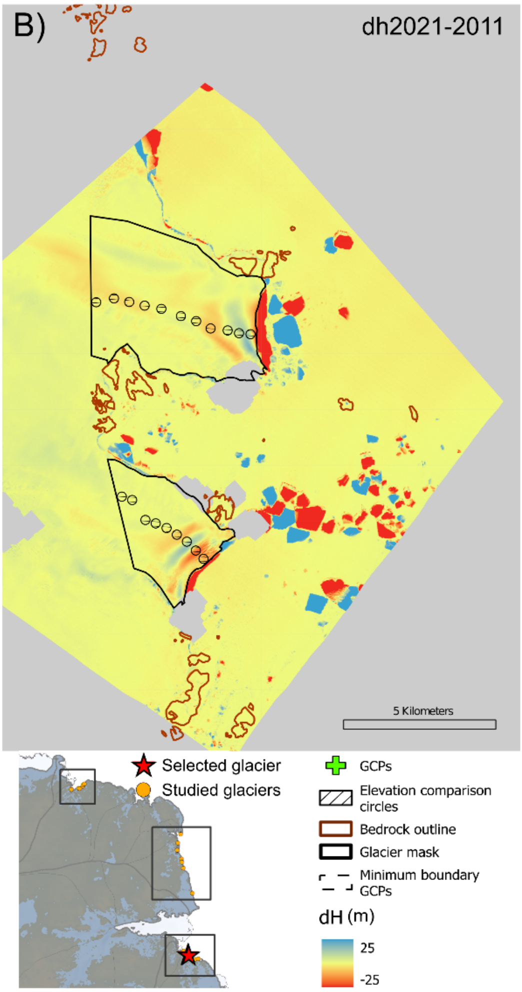

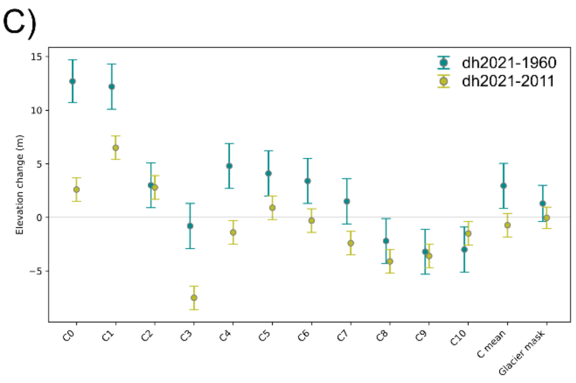

D)

Elevation comparison area	dh2021-1960 (m)	err2021-1960 (m)	dh2021-2011 (m)	err2021-2011 (m)
C0	12.7	2.3	2.6	1.5
C1	12.2	2.3	6.5	1.5
C2	3.0	2.3	2.8	1.5
C3	-0.8	2.3	-7.5	1.5
C4	4.8	2.3	-1.4	1.5
C5	4.1	2.3	0.9	1.5
C6	3.4	2.3	-0.3	1.5
C7	1.5	2.3	-2.4	1.5
C8	-2.2	2.3	-4.1	1.5
C9	-3.2	2.3	-3.6	1.5
C10	-3	2.3	-1.5	1.5
C-mean	3.0	2.3	-0.7	1.5
Glacier mask mean	1.3	2.0	-0.04	1.4

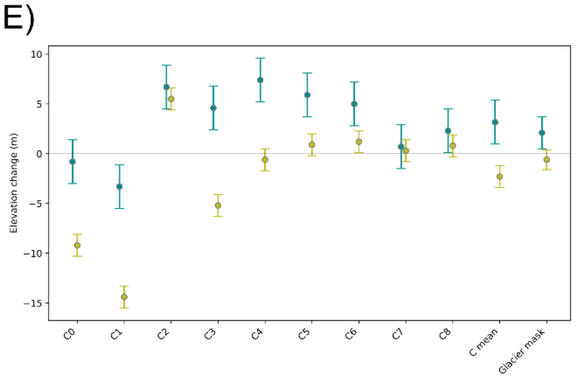

F)

Elevation comparison area	dh2021-1960 (m)	err2021-1960 (m)	dh2021-2011 (m)	err2021-2011 (m)
C0	-0.8	2.4	-9.2	1.5
C1	-3.3	2.4	-14.4	1.5
C2	6.7	2.4	5.5	1.5
C3	4.6	2.4	-5.2	1.5
C4	7.4	2.4	-0.6	1.5
C5	5.9	2.4	0.9	1.5
C6	5.0	2.4	1.2	1.5
C7	0.7	2.4	0.3	1.5
C8	2.3	2.4	0.8	1.5
C-mean	3.2	2.4	-2.3	1.5
Glacier mask mean	2.1	1.9	-0.6	1.4

G)

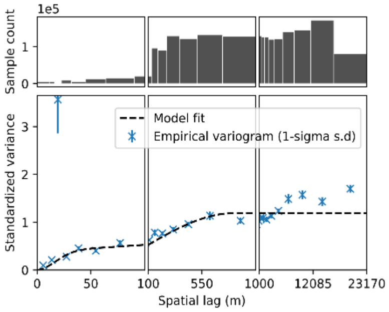

Fig. S18. Elevation changes at Flatnes and Hovde Glacier A) 2021-1960, B) 2021-2011. Elevation changes and associated errors of elevation comparison circle and the glacier area shown as C) and E) scatterplots and D) and F) tables for Flatnes Gl. and Hovde Gl., respectively. G) Spatial variogram of the historical DEM. Due to incomplete coverage of the 2021 reference DEM, the top left corner of A) shows the 1960 DEM in a black-to-white colorscale, which is included to show the full extent of the 1960 DEM.

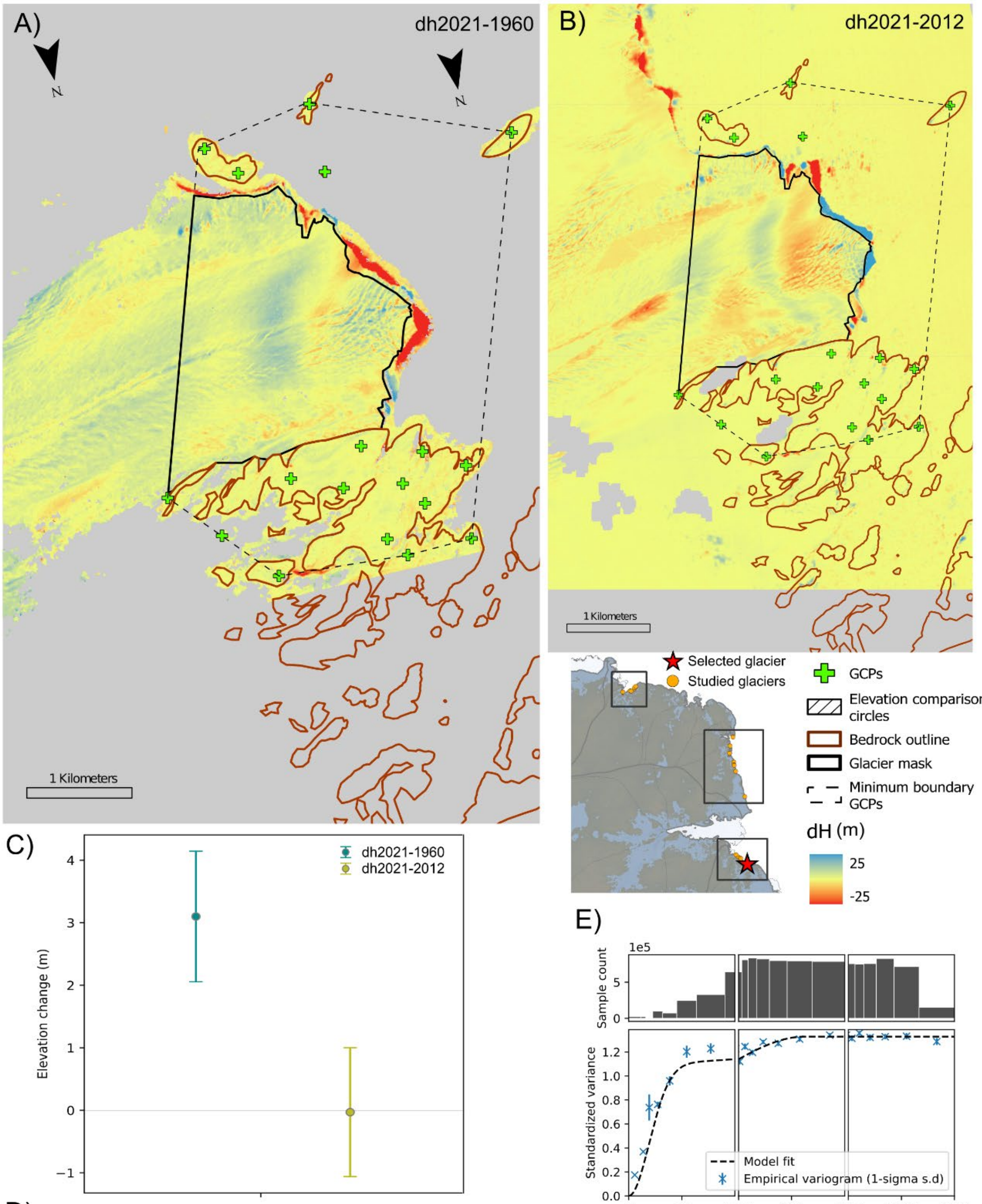

D)

Elevation comparison area	dh2021-1960 (m)	err2021-1960 (m)	dh2021-2012 (m)	err2021-2012 (m)
Glacier mask	3.1	1.0	-0.03	1.0

Fig. S19. Elevation changes at Brown Glacier A) 2021-1960, B) 2021-2012. Elevation changes and associated errors of the glacier area shown as C) scatterplot and D) table. E) Spatial variogram of the historical DEM

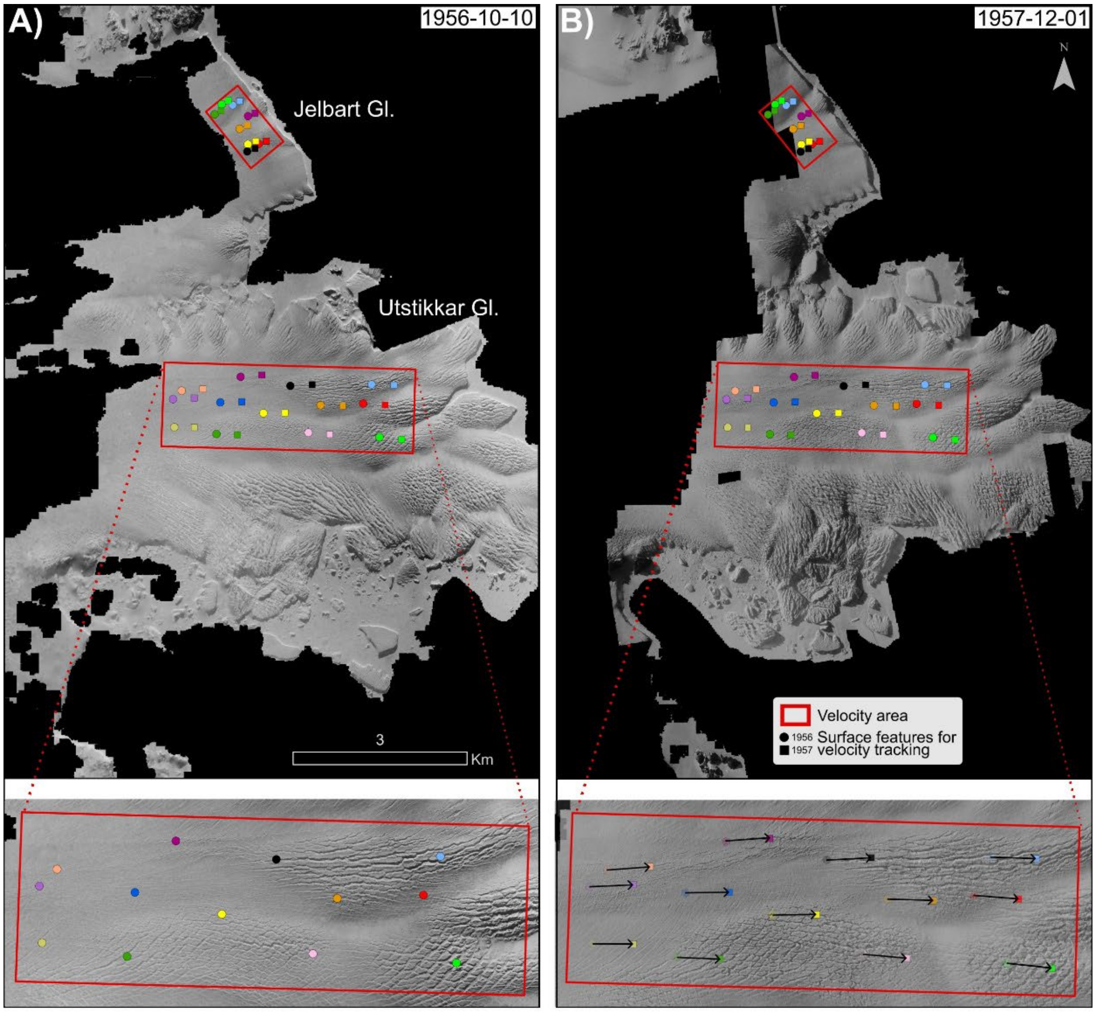

C) Utstikkar Gl.

Features (front to back)	Movement (m)	Annual velocity (m/yr)
f1	385	337
f2	385	337
f3	381	333
f4	383	335
f5	372	326
f6	378	331
f7	375	328
f8	366	320
f9	376	329
f10	366	320
f11	362	317
f12	363	318
f13	348	305
Mean	372	326

D) Jelbart Gl.

Features (NW to SW)	Movement (m)	Annual velocity (m/yr)
f1	130	114
f2	130	114
f3	141	123
f4	136	119
f5	148	130
f6	145	127
f7	139	122
f8	143	125
Mean	139	122

Fig. S20. A,B) Orthophoto mosaics of Jelbart and Utstikkar Gl. used for historical velocity estimations from images captured on 1956-10-10 and 1957-12-01. The velocity area (red box) is used for extracting ITS_LIVE annual velocity estimates and errors. Close up shows the 13 surface features used for historical velocity estimates of Utstikkar Gl. C) and D) Overview of all surface features used for calculating the historical velocities of Utstikkar and Jelbart Gl., respectively.

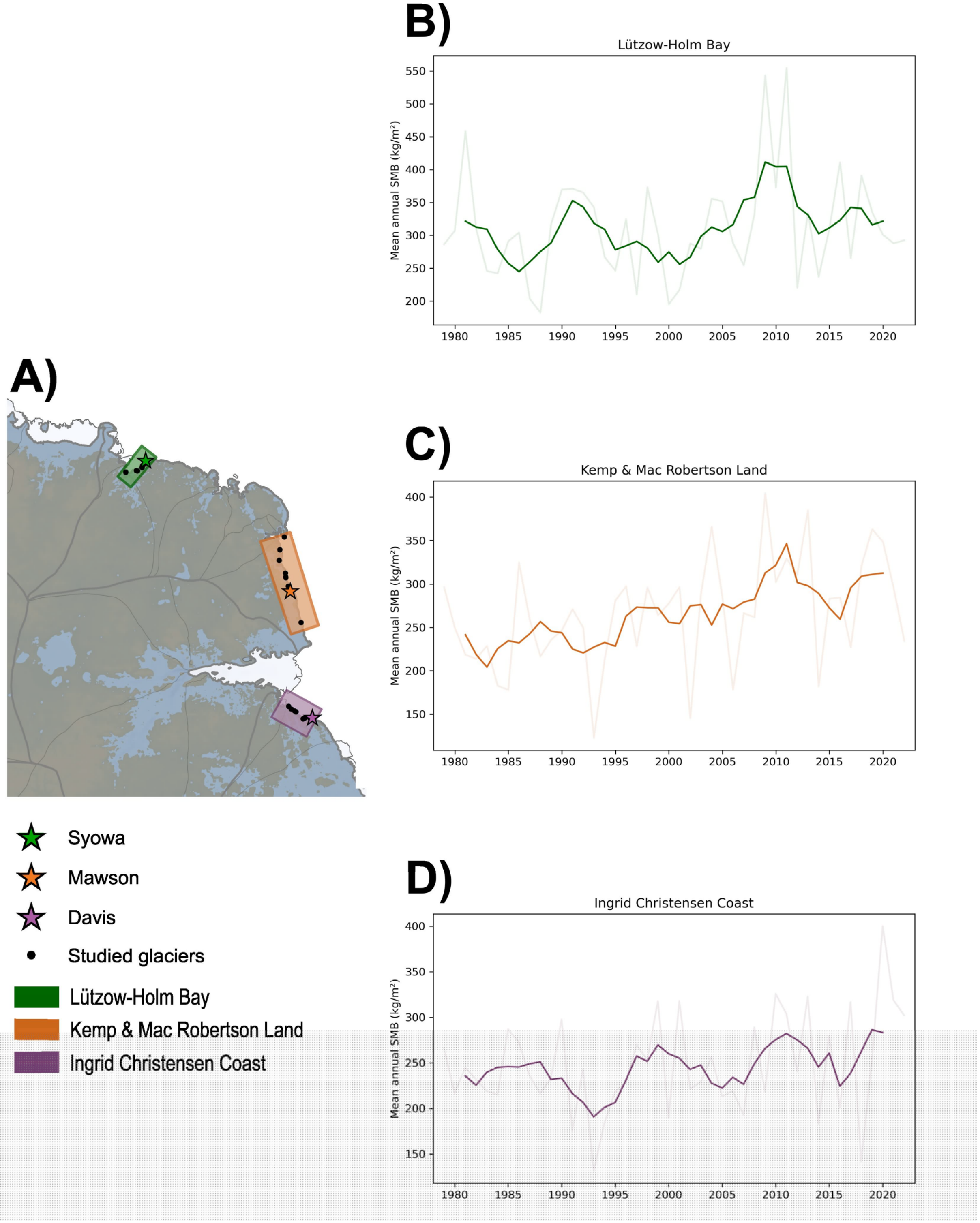

Fig. S21. A) Map showing the regions used for extraction of ERA5 snowfall and temperature data and RACMO SMB, as well as the location of Syowa, Mawson and Davis climate station. Panel B), C), D), show RACMO annual SMB (kg/m^2) plotted as 5 year moving average on top of annual average for B) Lützow-Holm Bay, C) Kemp and Mac Robertson Land, D) Ingrid Christensen Coast.

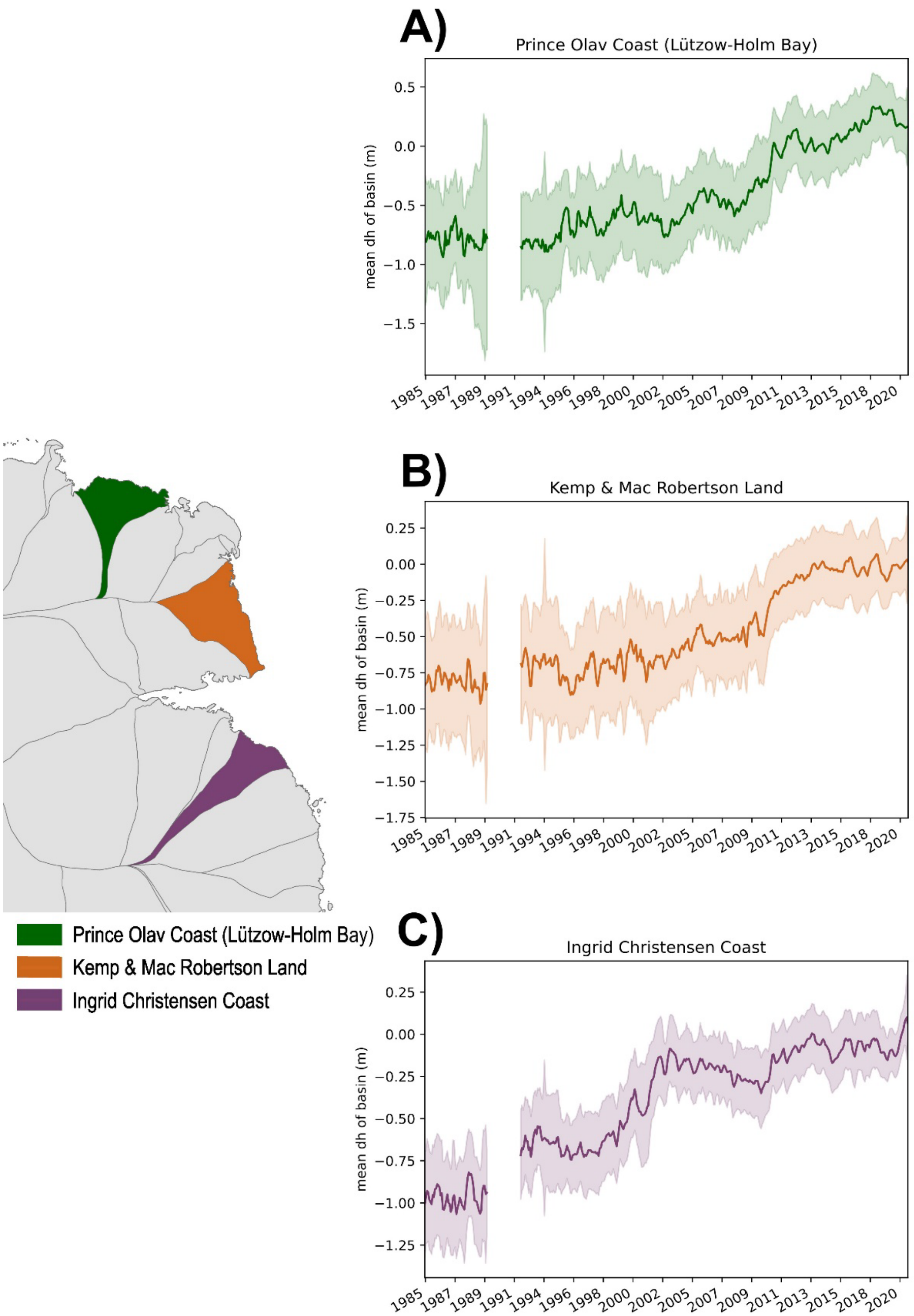

Fig. S22. Basin-wide mean dH from 1985 to 2020, with reference to 2013-12-16 of A) Prince Olav Coast (Lützow-Holm Bay), B) Kemp and Mac Robertson Land, C) Ingrid Christensen Coast. Data were obtained from satellite altimetry[3].

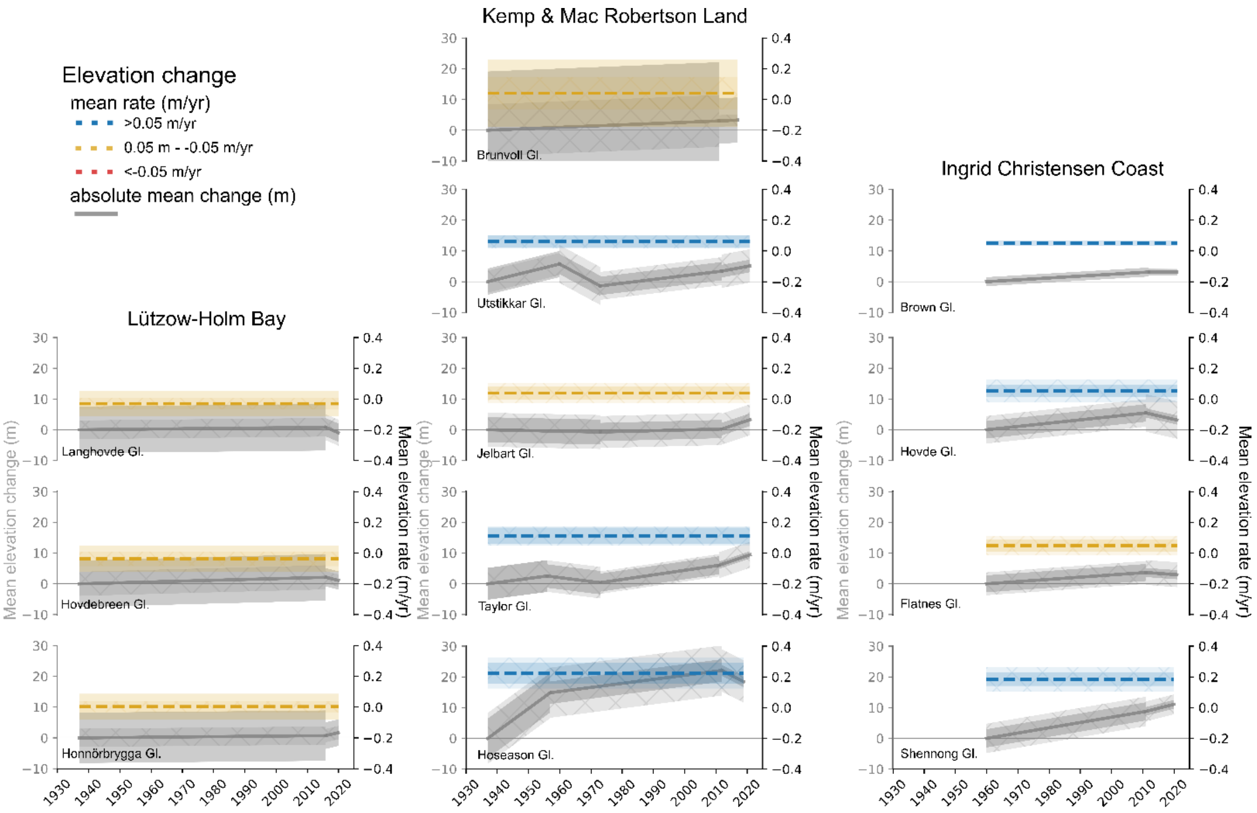

Fig. S23. Historical elevation changes of glaciers within each region (Lützow-Holm Bay, Kemp & Mac Robertson Land, and Ingrid Christensen Coast). The changes are presented as absolute values for each period and as the mean elevation rate for the longest available time span. The shaded area indicate the model uncertainty (1σ) of the produced DEMs (Data and Methods). The hatched shaded areas represent the sampling uncertainty (2σ + 1.5m) associated with the sensitivity of the calculated changes (Supplementary).

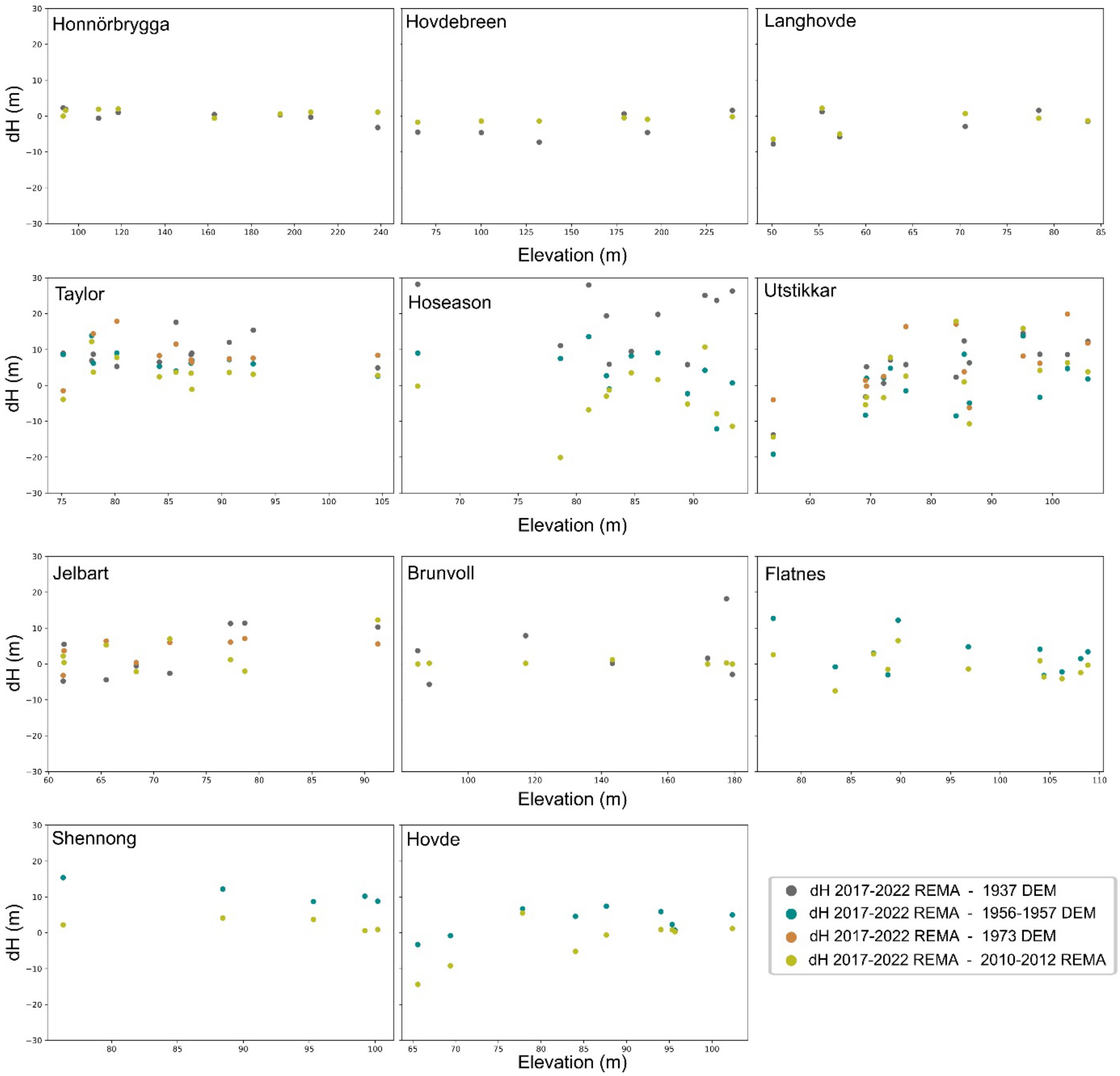

Fig. S24. Elevation change (dH) plotted against the elevation of all sampled circles for each glaciers. The dH of each epoch is calculated relative the most recent REMA DEM covering the area of interest between 2017-2022. The same REMA DEM is used to extract the glacier surface elevation (y-axis).

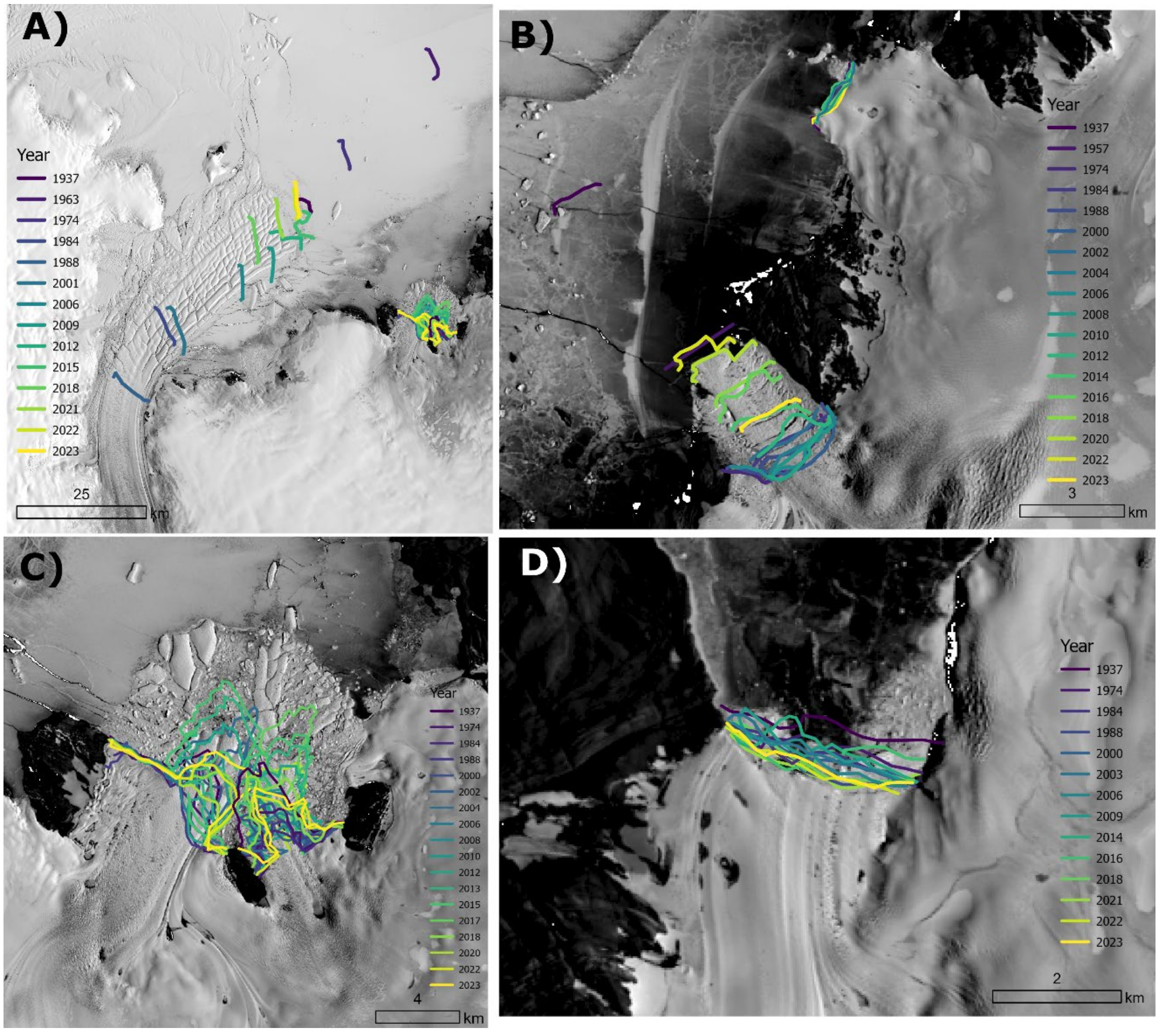

Fig. S25. Time series of frontal changes of glaciers in Lützow-Holm Bay. A) Shirase Glacier, B) Honnörbrygga and Hovdebreen Glacier, C) Telen and Skallen Glacier, D) Langhovde Glacier overlaid on Landsat scenes from 2018-2023

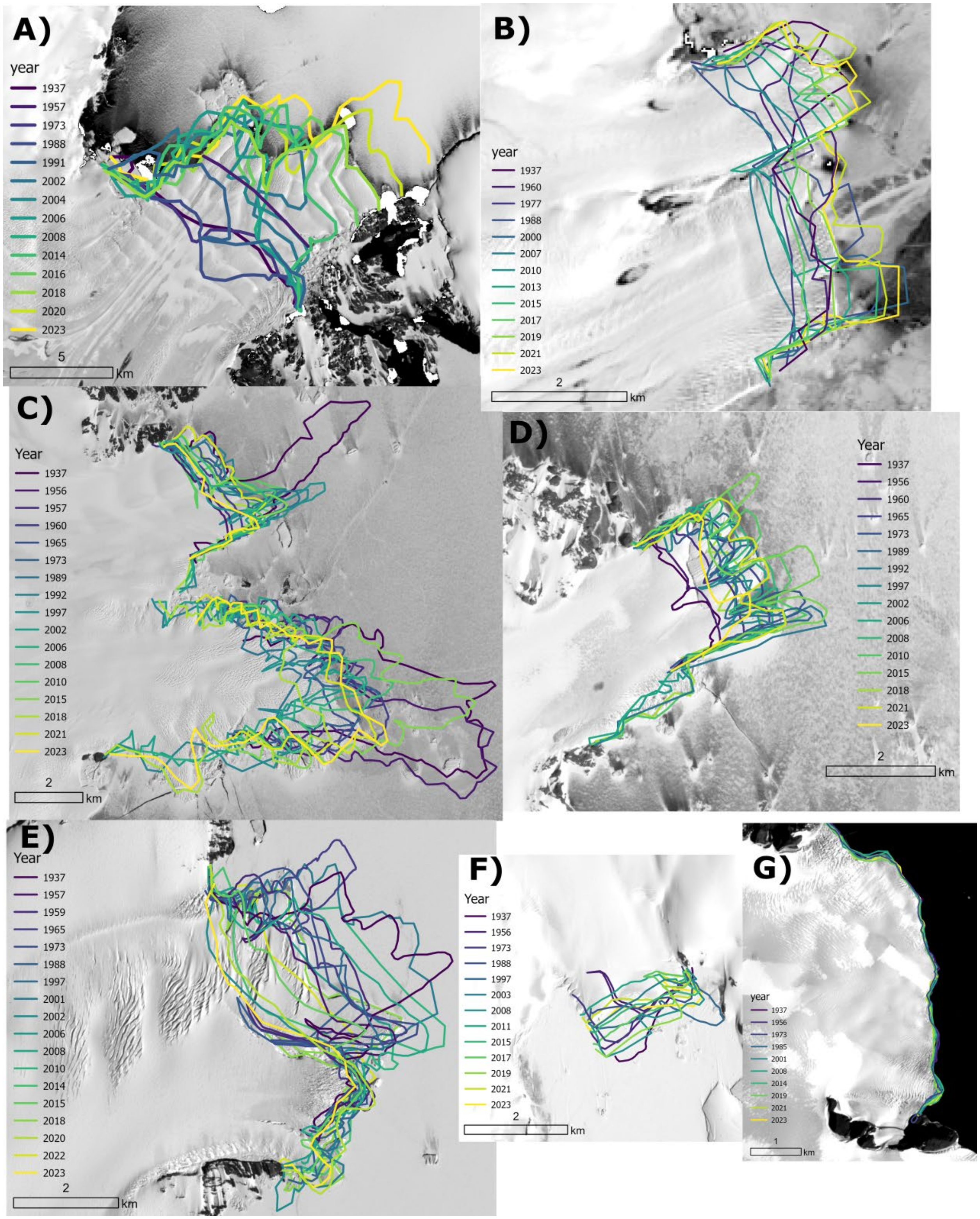

Fig. S26. Time series of frontal changes of glaciers in Kemp and Mac Robertson Land. A) Mulebreen Glacier, B) Forbes Glacier, C) Jelbart and Utstikkar Glacier, D) Taylor Glacier, E) Hoseason Glacier, F) Unnamed Glacier, G) Brunvoll Glacier overlaid on Landsat scenes from 2018-2023

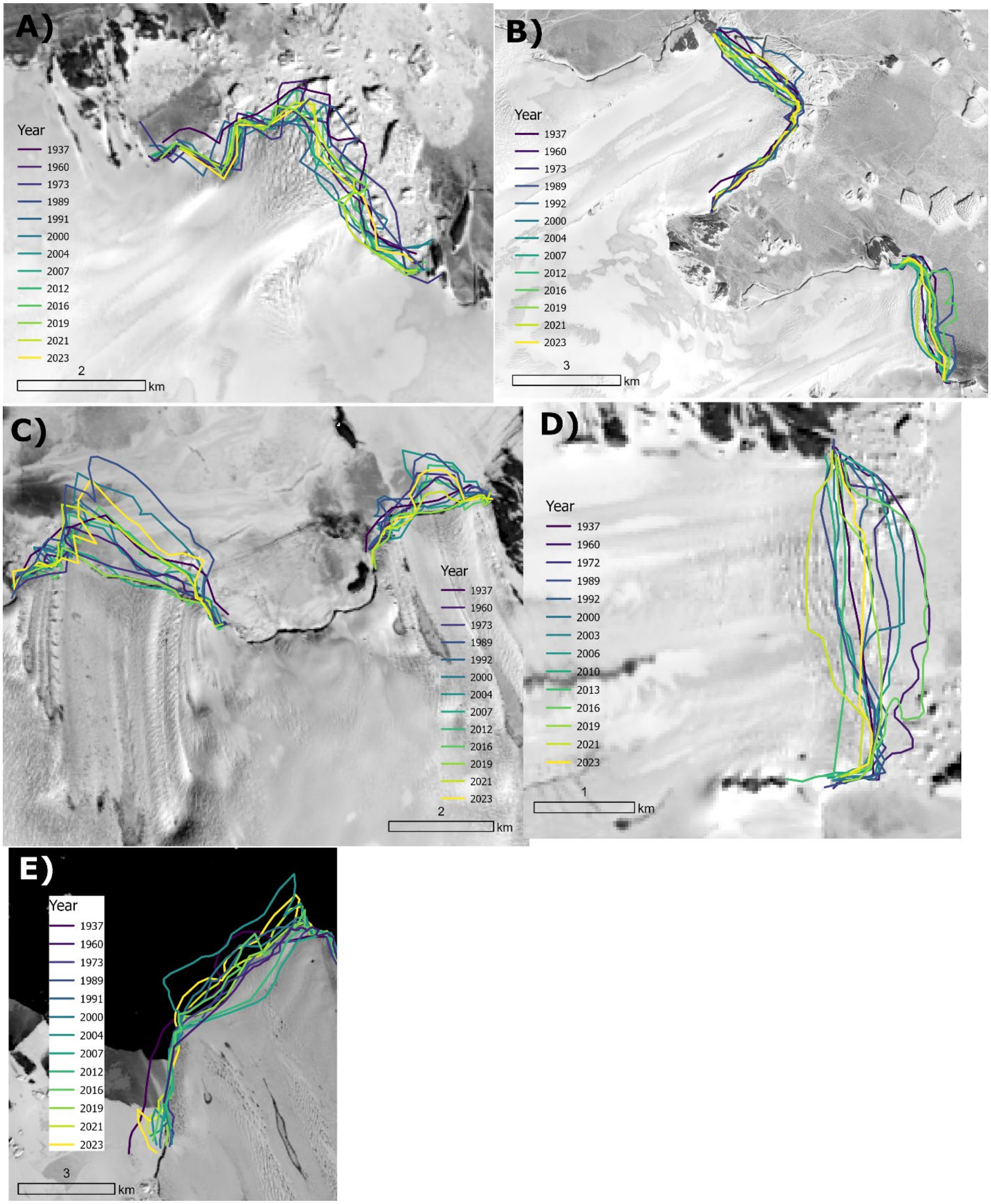

Fig. S27. Time series of frontal changes of glaciers along Ingrid Christensen Coast. A) Shennong Glacier, B) Flatnes and Hovde Glacier, C) Chaos and Brown Glacier, D) Dålk Glacier E) Sørdals Glacier overlaid on Landsat scenes from 2018-2023

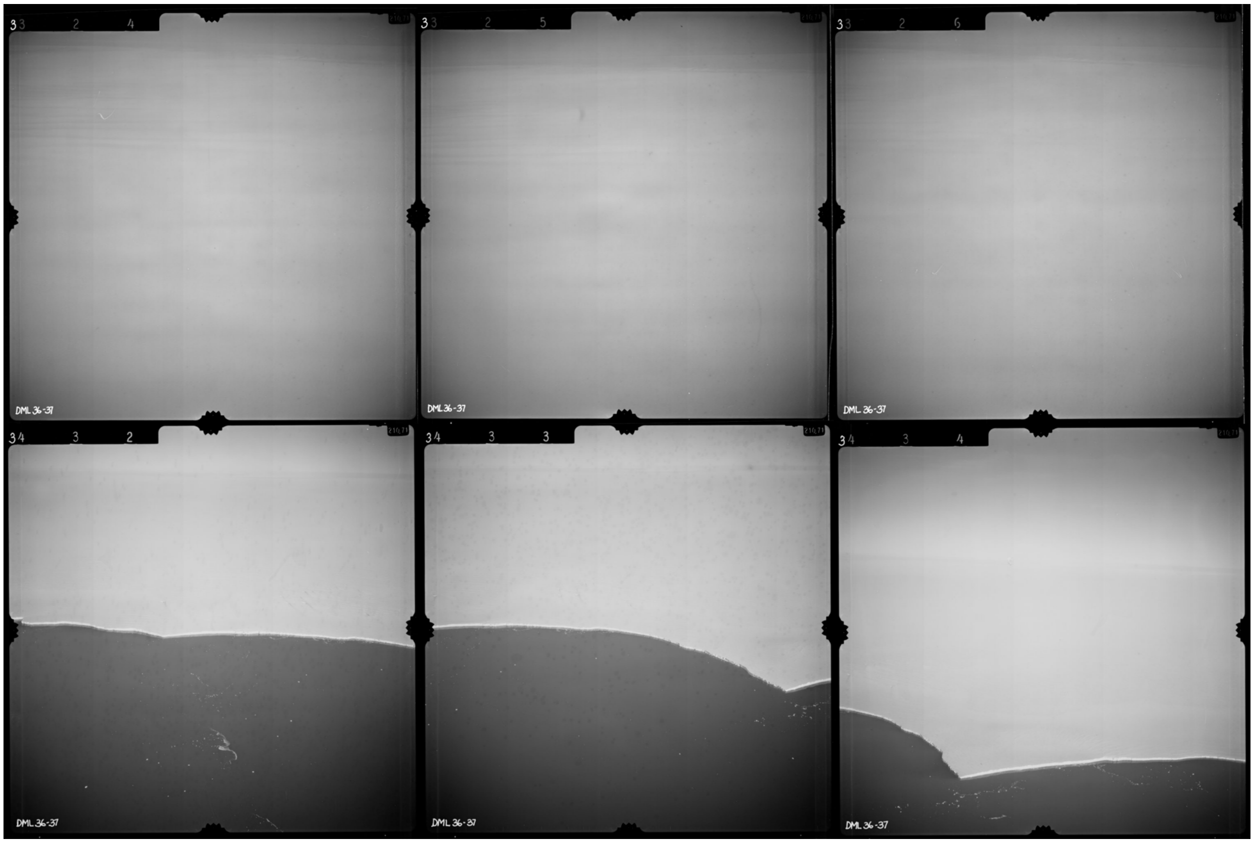

Fig. S28. 1937 aerial images of coastline in Dronning Maud Land. The images are captured far from the coastline, exhibit poor contrast difference over the ice and contains no visible bedrock for ground control point (GCP) selection.

References

1. Christensen, L. *Min Siste Ekspedisjon Til Antarktis 1936-1937* (Oslo, 1938).
2. Gudmundsson, G. H. Transmission of basal variability to a glacier surface. *Journal of Geophysical Research: Solid Earth* **108**, (2003).
3. Gardner, Alex, Fahnestock, Markand & Scambos, Theodore. MEaSUREs ITS_LIVE Antarctic Grounded Ice Sheet Elevation Change, Version 1. NASA National Snow and Ice Data Center DAAC https://doi.org/10.5067/L3LSVDZS15ZV (2023).
